# Oilbirds performing non-seasonal intra-tropical migrations reduce flight effort compared to individuals that frequently return to the colony roost

**DOI:** 10.1186/s40462-026-00686-2

**Published:** 2026-07-28

**Authors:** Gustavo Alarcón-Nieto, Adriana A. Maldonado-Chaparro, Carlos Bosque, Franz Kümmeth, Kamran Safi, Martin Wikelski

**Affiliations:** 1 Department of Migration, Max Planck Institute of Animal Behavior, Radolfzell, Germany; 2 Department of Biology, University of Konstanz, Konstanz, Germany; 3 Department of Biology, Universidad del Rosario, Bogotá, D.C Colombia; 4 Departamento de Biología, Universidad Simón Bolívar, Caracas, Venezuela; 5 e-obs GmbH, Grünwald, Germany

**Keywords:** Colonial birds, Central place foragers, Movement effort, Intra-specific variation, Movement strategies, Oilbird, Site fidelity

## Abstract

Individual behavioural variation is a driver of population dynamics and key for the adaptation and persistence of species. In colonial species, individuals share the same ecological niche and aggregate in space, but they might use different foraging strategies and incur differential costs. Using location and accelerometer data from 21 adult oilbirds (*Steatornis caripensis*), which are colonial frugivores, we characterized nocturnal movement patterns and measured total nightly flight time, as a proxy of movement effort, for different movement strategies during their non-breeding period. We collected location and accelerometer data using 22 g loggers (e-obs GmbH; Munich, Germany), between August 22nd, 2008 and February 14th, 2009. The tracked oilbirds displayed three distinct movement strategies for the entire sampling period: Intra-tropical migrants (ITM), which performed roost switches that lasted between 17 and 73 continuous days, consistent with partial nomadic migrations rather than seasonal movements; venturers, which performed long-distance excursions but returned to the main cave within 3 days, and roost-faithful individuals that never performed such excursions and never switched roosts. Among the three strategies, we estimated that ITM showed a lower movement effort, based on total nightly flight time, throughout the sampling period. Despite travelling long distances, ITM birds reduced overall flight effort by day-roosting near foraging sites, minimizing the need for long commuting flights. The oilbirds showed site fidelity to multiple foraging areas, without among-individual spatial overlap. These findings give insights into how behavioural flexibility could facilitate resource partitioning and coexistence in this colonial species, as well as the consequences of inter-individual variation for adapting to habitat change. Such information is relevant to evaluate the role of oilbirds as ecosystem service providers and habitat connectivity.

**Supplementary Information:**

The online version contains supplementary material available at 10.1186/s40462-026-00686-2.

## Background

Behavioural variation among individuals within a population shapes ecological interactions and population dynamics and is key for the adaptation and persistence of species [1]. While individuals of the same species share the same fundamental niche with similar ecological requirements, different behavioural strategies can emerge and result in substantial differences in space and resource use [2, 3], energy expenditure [4–6] and ultimately fitness [6]. In the context of foraging, individuals are, according to the optimal foraging theory and the principles of ideal free distribution, predicted to adjust their movement patterns to balance energy intake against foraging effort [7, 8], in response to their internal state, the environmental conditions and competition for resources [9–11]. While benefits are expected to ultimately offset movement effort, the effort itself constitutes a key axis of individual variation, with potentially broad ecological consequences (e.g [6, 12]). However, our knowledge of the differences in effort associated to behavioural strategies adopted by different individuals remains limited in free-ranging animals [7]. Documenting and quantifying such variation in movement strategies and the associated effort provides a first step toward understanding how populations composed of behaviourally distinct individuals may respond to environmental change. Such knowledge is also important for predicting how individuals might mitigate potential competition or disturbance through differences in space use rather than direct interactions.

Inter-individual differences in foraging strategies are particularly important in colonial species, where individuals commute from shared roosts or breeding sites to their foraging areas. In many colonial central-place foragers, larger colonies are associated with larger foraging ranges [13–15]. Higher densities near the colony can lead to rapid local resource depletion [14, 16], requiring longer commuting distances, and increased movement effort for at least some individuals. In such systems, movement decisions are expected to reflect a trade-off between commuting costs and access to profitable foraging sites. Theory predicts that individuals may distribute themselves in space in ways that reduce interference and exploit multiple patches rather than concentrating near the colony [8, 17, 18]. Empirical work in seabirds [1, 13, 19–22], colonial kestrels [6], and bats [2, 23, 24] have shown consistent individual differences in commuting distances, spatial segregation of foraging areas, and site fidelity that can reduce overlap and competition, but can also impact the balance between food intake and movement effort at the individual level. Against this general backdrop, colonial species with specialised diets, where resource differentiation is limited, provide an opportunity to examine how individuals balance range-wide movements with alternative roosting strategies, and how these differences translate into variation in movement effort, providing indirect insight into the drivers and consequences of individual strategies.

Individuals with consistently longer commuting distances or increased movement effort incur elevated costs, which they must offset through sufficient resource gain or other benefits. In colonial cormorants, commuting to foraging sites has been identified as the most energetically costly part of movement [25], suggesting that individuals foraging closer to the colony could reduce flight costs substantially [26]. However, the higher density of individuals near a colony can increase search effort compared to conspecifics foraging at the periphery, as observed in black-legged kittiwakes [15]. Nevertheless, higher energy expenditure is not necessarily disadvantageous. In lesser kestrels, a colonial raptor, individuals adopting more mobile foraging strategies (i.e. more searching flights, less perching) experience higher reproductive success despite greater energetic expenditure, suggesting that increased cost can be offset by improved resource acquisition or other fitness benefits [6]. While these examples illustrate that variation in movement effort can be associated with different ecological benefits across systems, the generality of these trade-off patterns remains uncertain. Assessing how different movement strategies relate to movement costs in other species and foraging guilds is key to understanding how individuals interact with their environment and the scope for the emergence of multiple strategies to coexist within populations.

We investigated individual variation in the nocturnal flights of oilbirds (*Steatornis caripensis*) and their movement strategies, to gain insight into how a colonial species with an obligate frugivorous diet may cope with the costs of commuting as a central-place forager. Oilbirds colonially roost and breed in caves in northern South America [27–30], and are known to perform a post-breeding intra-tropical migration [27], and to frequently day-roost in forests or alternative caves [28]. Oilbirds provide a suitable study system to compare movement effort because, in addition to their movement capabilities, they use flapping flight as their only mode of locomotion. Flapping causes a clearly detectable signal on accelerometer (ACC) sensors, allowing measurement of the timing and duration of each movement bout without having to account for other locomotor modes such as soaring, walking, or diving, which can complicate similar analyses in many other colonial species. Oilbirds are strictly nocturnal and typically commute from the roost to the foraging areas where they perform short foraging flights, ending the night with a return flight to the roost [28]. The difference between flying and roosting periods can be detected using loggers attached to the birds [28]. This allows estimating flight duration and assessment of among-individual differences in flight behaviour to compare movement effort as a proxy for cost of transport associated with different nocturnal strategies. In this study, we used ACC and location (GPS) data to quantify individual variation in nocturnal movement patterns and non-breeding movement strategies. Oilbirds are central-place forages, which means that they engage in commuting flights to reach their foraging grounds. Because commuting flights account for a large part of the foraging movement energy budget [25, 31], we expect that: (1) if individuals repeatedly use the same foraging areas (i.e. site fidelity), those that perform longer commutes experience higher season-wide movement effort; (2) and that roosting near the foraging areas, as reported by Holland et al. [28], could result in strategies to reduce the frequency and length of commutes. We discuss these results in the context of the observed variation and its consequences for space use, their potential for resource partitioning and the role of oilbirds in the ecosystem.

## Methods

### Tracking data

We included data from 21 oilbirds tagged at Cueva del Guácharo in Venezuela, caught with a 12 m long by 2 m-high mist net placed close to the entrance of the cave. We caught birds on August 21st, 2008, as they departed from the cave after dark. The birds were processed immediately after capture. Individuals included in this study were adults, identified by the wear of wing and tail feathers, minimum wing length of 308 mm [30] and weighing above 400 g. Individuals that did not match these characteristics were immediately released. Those that were selected were fitted with 22 g battery-powered GPS-ACC loggers (e-obs GmbH; Munich, Germany) attached on their back, using Teflon ribbon crossover wing harnesses [32], adding a maximum of about 5% of the birds’ body weight, which is commonly accepted by the ethics standards [33, 34], although more recent studies suggest lower loads [34]. Although, the effects of tagging [35] could be a source of bias in this study, we do not have any evidence to believe that our observations do not represent the natural behaviour of the birds. We selected large adult individuals, while choosing the smallest biologging devices available given the project demanded operational duration and the necessity for remote data transmission to obtain the data in the rainforest, both of which combined allowed us to not exceed this (upper) limit. Without devices recording position through time, it is simply impossible to have a quantitative benchmark for birds that are nocturnal and forage across vast areas in the tropical lowland rainforest. All birds were released between 10 and 60 min later and all flew off well, either to the forest or into the cave. Due to the low chances of recapturing the birds, it was not possible to retrieve the tags once data collection was over. Data were retrieved using a handheld base station that detected the loggers at a frequency of 868.3 MHz, during late-afternoon walks inside the main chamber of the cave. The tracking period was between August 22nd, 2008 and February 14th, 2009, which roughly represents the non-breeding season of the species [27]. We collected GPS data during one night every week, getting a single location every hour between 17:30 h and 05:30 h, local time. On a nocturnal cave-roosting bird, this setup allowed for prolonging the battery life of the loggers and extending the sampling period. For ACC, we recorded data at 17.5 Hz in two-second bursts every two minutes. We used single-axis ACC data to assess the vertical acceleration of the birds and differentiate between flying and roosting periods, expanding previous observations [28] with a quantitative approach to describe and compare nocturnal movement patterns. For each two-second burst, we calculated the maximum-minimum difference in the raw ACC values and assigned it to one of three categories: (1) Flying/flapping (max-min ACC difference > 600), (2) Roosting (max-min ACC difference < 599). We evaluated the robustness of this classification, through a sensitivity analysis, by varying the flying/roosting threshold between max-min ACC difference 400 and 1000, by 25 increments, and quantified the agreement with the classification using the selected value (max-min ACC difference = 600), which remained above 95% throughout the range (Fig. S1). Using this classification, we then estimated the duration of each behavioural bout by grouping consecutive two-second bursts of the same category and extracted the timestamp of the first and last burst. To minimize noise in the data, we excluded changes in behaviour lasting only a single two-second burst.

We used the flying/flapping bursts for each individual and night (17:30 h − 06:30 h local time) to calculate: (1) Start of nightly activity: the timestamp of the start of the first flight, (2) Total active time per night: the time difference between the beginning of the first flight and the end of the last flight, (3) total nightly flight time: the sum of the duration of all flights of an individual in one night, (4) Commute time: duration of the early-night commute-out from roost to foraging areas, and the commute-in before sunrise from the foraging areas to a roost, and (5) Number and duration of foraging flights, defined as all the flights between the end of the outbound commute (after dusk/roost to foraging areas) and the start of the inbound commute (before dawn/foraging areas to roost).

### Nocturnal movement patterns

**Fig. 1:**
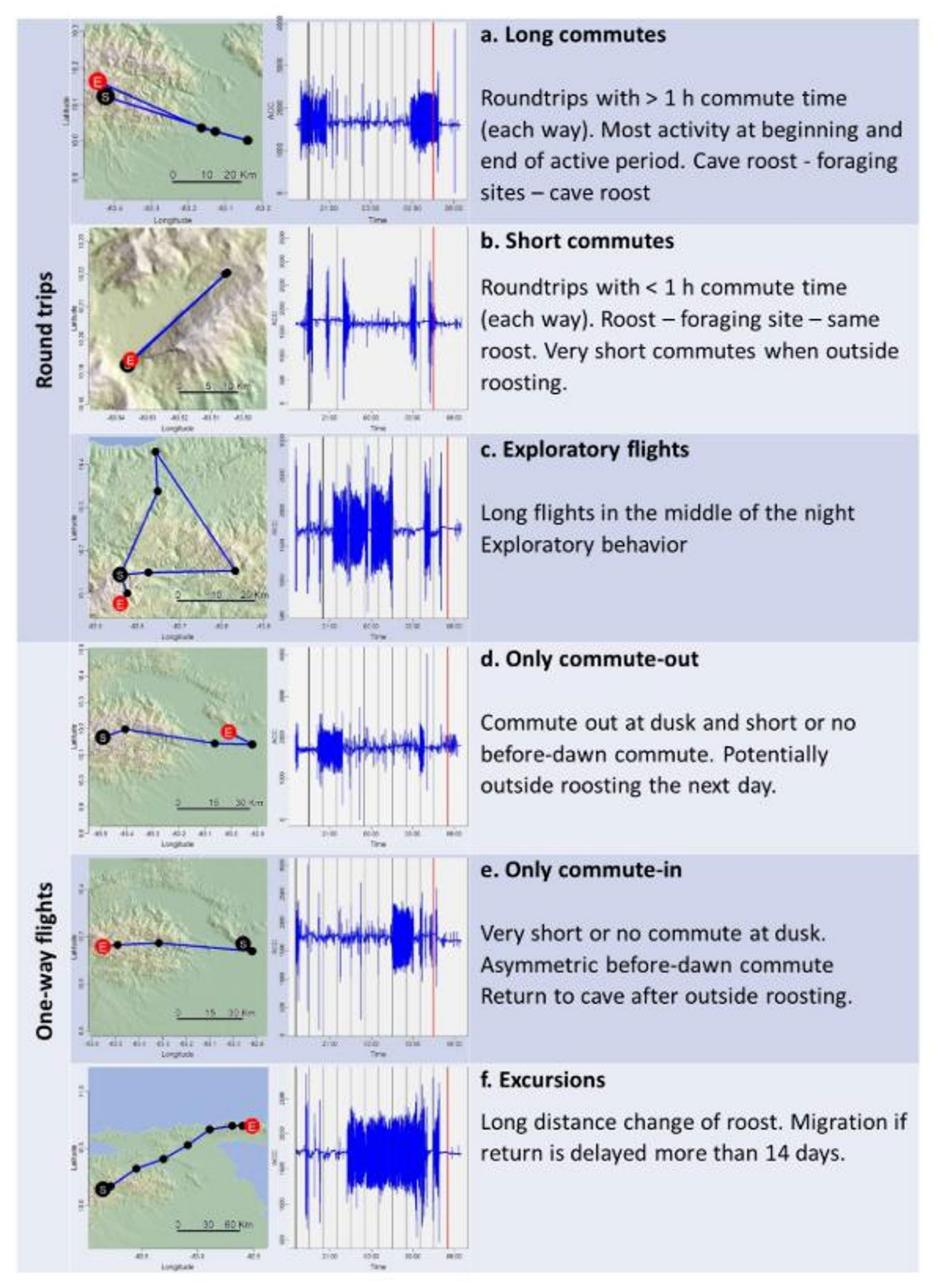
Nocturnal movement patterns. Classification based on the temporal symmetry of the individual’s flight activity. Round-trips (**a**-**c**) are characterized by symmetric activity and return to the same roost, and one-way trips (**d**-**f**) have asymmetric activity, with a change of roost. On the map, black dots with an S show the first GPS fix and red dot with E, the last GPS fix of the night

Since location data based on GPS (Global Positioning System) was only collected during one night every week, we used nights with both GPS and the information extracted from the ACC data to quantitatively characterise nocturnal movement patterns based on the temporal symmetry of the flying activity (round-trip or one-way flights) and the length of the commute flights (see Fig. 1). We used a quantitative approach which allowed us to classify each night with only ACC data into one of the six nocturnal movement patterns (Fig. 1) as follows: First, we quantified the symmetry of the flights for each individual, by dividing the total active time per night (see ACC data methods) into quarters (Q1 – Q4) and calculated the flight time for each quarter. Symmetric nights represent round-trips from and to the same roost (Fig. 1a-c) and exhibited a ratio between 0.66 and 1.5 of flight time between the first and second halves (Q1 + Q2: Q3 + Q4), Asymmetric nights represent one-way flights (changes of roost; Fig. 1d-f), exhibited a ratio below 0.66 or above 1.5 of flight time between the first and second halves (Q1 + Q2: Q3 + Q4). Commute flights correspond to the longest of the first two flights in Q1 (commute-out) and the longest of the last two flights in Q4 (commute-in). Among the round-trips, we used the distribution of flight time across the four quarters to differentiate: Long commutes (Fig. 1a): highest activity at the beginning and end of the active period (Q1 and Q4), with flight time in Q1 + Q4 larger than the population mean – 0.25 SD (i.e., 3632 s per commute); short commutes (Fig. 1b): flight time in Q1 + Q4 less than the population mean – 0.25 SD (i.e., 3632 s per commute); exploratory nights (Fig. 1c): high amount of flight time in the middle of the night (Q2 + Q3), regardless of the commute time, possibly related to the exploration and use of multiple foraging sites in one night. One-way trips were also differentiated into three patterns: only commute-out (Fig. 1d): highest activity in Q1, which represent flight from the roost with no return; only commute-in (Fig. 1e): highest activity in Q4, which represent flights from the foraging areas to the roost, likely return to a cave after day-roosting outside; and excursions (Fig. 1f): nights with flight time higher than individual’s mean + 2SD, calculated across all recorded ACC nights. We performed sensitivity analyses for the thresholds used to classify short/long commutes on nights with roundtrips (Fig. S2), and excursions (Fig. S3) and found our classification is robust, with consistency above 95% for both thresholds.

### Movement strategies

We classified individuals into one of three movement strategies that describe their movement during the whole sampling period. This classification is based on the nocturnal movement patterns used and the number of days the individuals roosted near or at the foraging areas before returning to the cave. We inferred that a bird roosted outside a cave at the foraging site, if after a night with only commute-out (Fig. 1d), the length of the commute-out, and the commute-out of the following days was equal or shorter than the individual’s mean foraging flight. The duration of this behaviour was measured as the number of consecutive days until an only commute-in night (Fig. 1e) was recorded, indicating that the birds could have returned to the cave. We defined the *intra-tropical-migratory* (*ITM)* strategy, for individuals that performed excursions (Fig. 1f) and then spent at the least two continuous weeks at alternative roosts, away from the tagging site (i.e. alternative roosts used for 17–73 continuous days, 66–324 km away from the tagging site, Alarcón-Nieto et al. in prep). This is consistent with partial nomadic intra-tropical migratory movements [36], likely related to resource-tracking rather than strict seasonal breeding/non-breeding migration. Individuals that performed excursions but only stayed outside three or fewer days before returning to the cave belong to the strategy *Venturers*. The last strategy, defined as *roost-faithful*, represents individuals that never exhibited excursion flights.

### Movement effort

We used the total nightly flight time as a proxy for movement effort. Specifically, we summed the duration of all flapping flight bouts (flying/flapping bursts) per night, including commutes to and from the roost and all other flights (foraging, excursions, etc.). Although our ACC data are restricted to a single axis and relatively low sampling frequency, previous studies using higher-resolution tri-axial accelerometers show that dynamic body acceleration and related activity metrics (including dynamic body acceleration, DBA, overall dynamic body acceleration, ODBA, and vectorial dynamic body acceleration, VeDBA) are tightly correlated with daily energy expenditure [37–39]. Across locomotor modes, mean DBA explains a large proportion of the variation in daily field metabolic rate, and DBA-based models often perform as well as or better than simple time-budget approaches [37–39]. In Australasian gannets, models that combine mean VeDBA with total distance travelled provide the best predictions of energy expenditure, indicating that accelerometry-derived activity covaries with overall movement distance and cost during commuting trips [38]. Given this evidence, and the fact that oilbirds use flapping flight as their only locomotor mode, with wingbeats producing a detectable accelerometer signal, we consider total nightly flight time a biologically meaningful proxy of relative movement effort that is likely related to energy expenditure, while deliberately referring to it as effort to avoid over-interpreting our coarser ACC data and the possibility that other activities such as hovering in fruiting trees might add noise to the measurement of movement effort [40, 41].

### Statistical analysis

To evaluate consistent individual differences in movement effort while accounting for the non-independence of sequential tracking data, we estimated the repeatability (R) of nightly flying activity using linear mixed-effects models (LMM) via the nlme package in R [42]. We fitted total nightly flight time (a proxy for movement effort) as the response variable and individual identity as the random effect using a Gaussian error distribution with an identity link function. We addressed temporal autocorrelation driven by consecutive tracking nights by incorporating a continuous first-order autoregressive structure (AR(1)) into the model’s residual error matrix, grouped by individual bird (corARrpt1 structure). Time steps were designated using the actual days elapsed since each bird’s initial tracking night to properly account for irregular sampling gaps. Gross repeatability was calculated as the proportion of total variance explained by the among-individual variance component. To generate robust uncertainty estimates around R, we implemented an individual-level non-parametric bootstrap (*n* = 1000 iterations) with replacement, calculating 95% confidence intervals from the empirical distribution of converged iterations.

To examine whether different movement strategies were linked to differences in movement effort, we used the package lme4 v. 1.1–35.3 [43] to fit a LMM with a Gaussian error structure, with total nightly flight time as the response variable, behavioural strategy as the fixed effect, and individual identity as the random effect. Then, we performed a post-hoc multiple comparison test (Tukey HSD) using the R package emmeans 1.10.3 [44], and calculated the p-values with the package lmerTest v. 3.1-3 [45]. All models were run in R v. 4.3.3 [46].

## Results

**Fig. 2:**
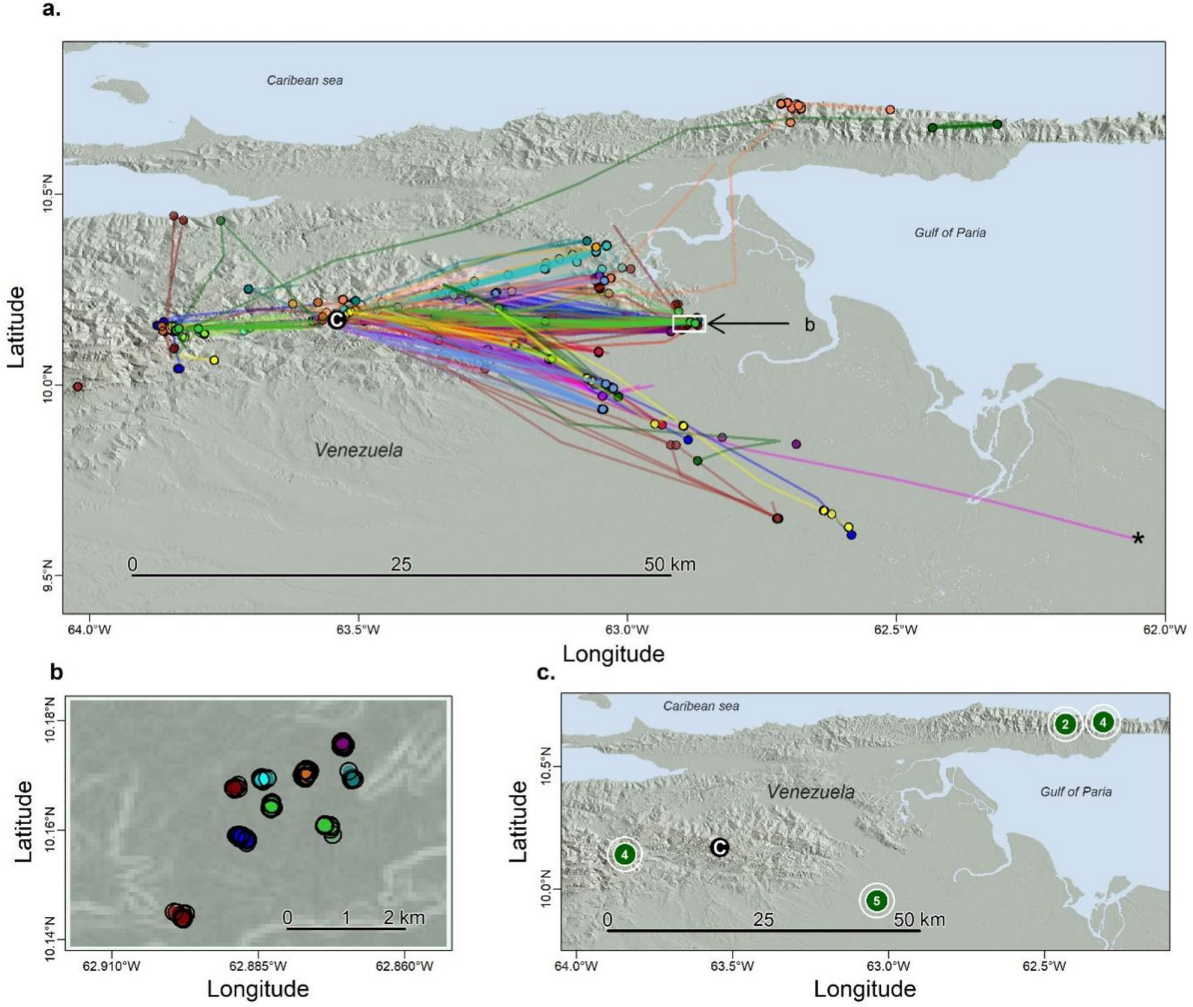
Movement of oilbirds. **(a)** Tracks of 21 individuals during the entire sampling period (Aug 2008 - Feb 2009). Colours represent different individuals. Lines show movement between roosts and foraging areas and dots represent foraging locations. Black circle with C is the location of the tagging site. Asterisk (*) indicates that some distant locations were removed to simplify visualization. Black arrow shows location of map (b) (**b).** Individual segregation at the foraging areas. Coloured dots represent location of different individuals throughout the entire sampling period (Aug 2008 - Feb 2009) in a ca. 30 km^2^ area. **(c)** Site fidelity of a single individual to multiple foraging areas (Oilbird #89009, 84 GPS locations over 15 nights), the numbers in the green circles are nights (revisits) at each site. See maps for other individuals in supplementary materials (Fig. S4-S24)

For the 21 individuals in our study, we collected data on 296 GPS nights (mean ± SD = 12.25 ± 4.83 nights; range = 6–15 nights, one night per week, per individual, see Table S1) and 2178 ACC nights (mean ± SD = 90.7 ± 47.8 nights; range = 31–176 nights per individual). The GPS and ACC data showed that oilbirds used non-stop (commute) flights from the roost to the foraging sites (Figs. 1 and 2a). Nightly foraging locations, GPS fixes collected between the end of the commute-out flight and the start of the commute-in, showed no among-individual spatial overlap (Fig. 2b), throughout the entire sampling period. The mean minimum distance between two individuals was 4608.5 m (SD = 8249.5), 95% of the pair distances where larger than 100 m, and only two pairs were below 10 m, which is the mean error of the loggers (SD = 12 m). Individual oilbirds revisited multiple small-sized foraging areas (1–5 per individual), at varying distances from the main roost, as far as 297 km, reached in up to three nights of excursions (Fig. 2c, S4 - S24, Table S1).

ACC data showed that each night, oilbirds averaged 10.07 ± 1.47 h of active time (range 0–12.37 h), with large individual differences in total nightly flight time (219.2 ± 14.81 min). Commute flights from the roost to the foraging areas had a mean of 54.9 ± 44.62 min (range = 2–498 min). At the foraging areas, oilbirds performed on average 4.6 ± 2.4 flights per night (range 0–16) with a mean duration of 12.1 ± 8.5 min (range = 10.1–27.7 min).

### Nocturnal movement patterns

**Fig. 3:**
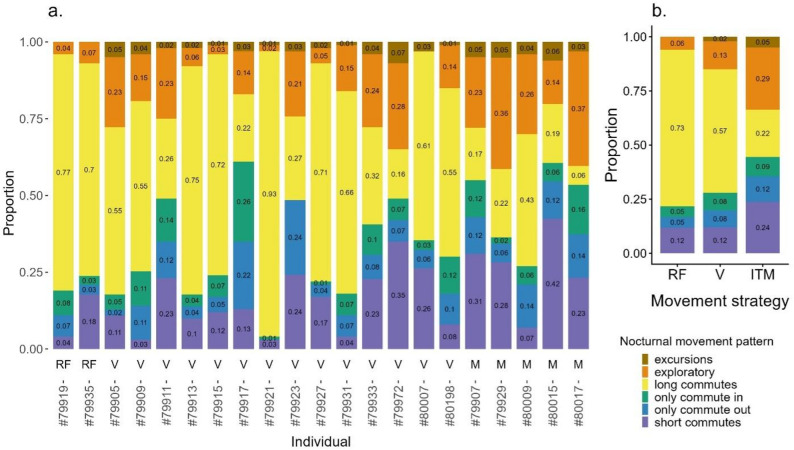
Nocturnal movement patterns. **(a)** Individual differences in the proportion of use of nocturnal movement patterns. Individuals are grouped by movement strategy. RF: Roost-faithful, V: Venturers, Intra-tropical migrants: ITM. **(b)** Comparison among movement strategies

Except for excursions, all individuals used all nocturnal movement patterns (Fig. 3a). Round-trips with long commutes accounted for 43.8% of the sampled nights, whereas round-trips with short commutes and exploratory flights occurred on 15.8% and 16.7% of the nights, respectively. One-way trips with only outbound commutes were recorded on 8.5% of the nights, while 7.9% represented return flights. The remaining nights (2.7%) were excursions, which represent within individual long-distance changes of roost.

The proportion of use of each movement pattern varied widely among individuals. This variation ranged from individuals that performed long commute round-trips almost exclusively to individuals with higher proportions of short commutes (Fig. 3a).

### Movement strategies

Two individuals were classified as roost-faithful. They never performed excursions and day-roosted away from the cave 5% of the days. Among the remaining individuals, 14 were described as venturers, which performed excursions but returned to the cave within three days. Venturers roosted at foraging sites up to 25% of the time. The remaining five individuals were classified as ITM and shifted roosts to stay closer to the foraging areas between 17 and 73 continuous days, which accounts for up to 42% of the days (Table S1). This results in a reduced proportion of nights with long commutes (Fig. 3a) for ITM individuals.

### Movement effort

**Fig. 4:**
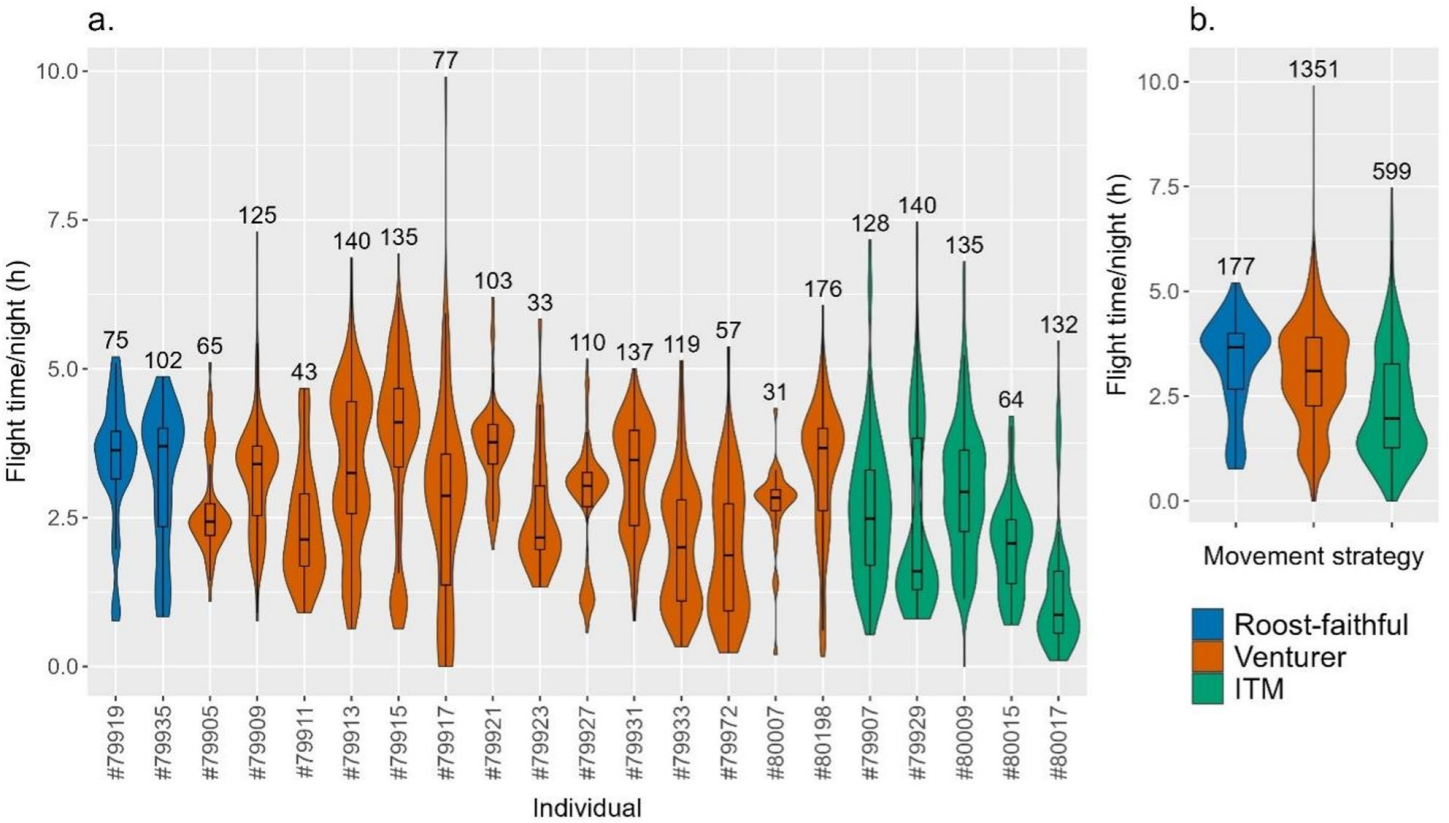
Total nightly flight time. **(a)** Individual differences in nightly flight time grouped by movement strategy. **(b)** Comparison of mean flight time among movement strategies. Box-plots centred at the mean. Numbers on top represent number of nights with ACC data for each individual

We found high individual variability in total nightly flight time, ranging from 71.8 to 228.4 min (i.e., 1.2 to 3.8 h) (Fig. 4a). Total nightly flight time showed a moderate within-individual consistency, with a repeatability of *R* = 0.244 (95% non-parametric bootstrap CI: 0.115, 0.362). We detected a strong temporal autocorrelation between consecutive nights (Φ = 0.473), indicating that night-to-night variation in an individual’s flight duration was directly carried over from its behaviour on the preceding night. Overall, the individuals using the ITM strategy exhibited a significantly lower mean total nightly flight time during the sampling period (mean 2.26 ± 1.34 h, *N* = 599) compared to those using the roost-faithful (mean 3.28 ± 1.12 h, *N* = 177, B = 0.80, SE ± 0.37, t = 2.18, *p* = 0.04) and venturer strategies (mean 3.03 ± 1.24 h, *N* = 1351, B = 0.50, SE ± 0.23, t = 2.17, *p* = 0.04) (Fig. 4b). Post-hoc comparisons showed that the venturer and roost-faithful strategies resulted in similar movement effort (*p* = 0.37). ITM individuals had fewer nights with long commutes, and day-roosted more outside the cave, whereas roost-faithful individuals exhibited more nights with long commute round-trips (Figs. 3b and 4b).

## Discussion

Our study reveals individual variation in movement behaviour with marked differences in movement effort. We identified six nocturnal movement patterns (Fig. 1) and three movement strategies: ITM, roost-faithful, and venturer (Fig. 1b), and estimated the associated movement effort by accounting for total flight time per night (Fig. 4). Counter-intuitively, we found that ITM individuals, despite performing long-distance excursions, spent less time flying and thus had lower movement effort during the non-breeding season compared to roost-faithful and venturer individuals (Fig. 4b). This is explained by the fact that the time they spent away from cueva del guácharo, individuals often day-roosted close to the foraging sites, potentially in trees, reducing the frequency of costly commute flights. The tracked oilbirds exhibited site fidelity to multiple foraging areas (Fig. 2a, S4-S24) and we observed no spatial overlap among individuals at the foraging areas across a large area (Fig. 2b, Table S1). This suggests that, as in other colonial species [1, 22, 47], spatial segregation could facilitate resource partitioning in oilbirds.

Contrary to expectations for central-place foragers [15, 20], we found no evidence of a higher density of oilbirds near the cave nor overlapping space use at nearby foraging areas during the sampled period, which corresponds to a non-breeding stage. Despite our small sample size, the majority of the tracked individuals, with the exception of the roost-faithful, performed excursions that reached farther foraging sites. These long-distance movements and the associated locomotion effort could be the result of current or past intra-colony competition, as suggested for other colonial species [13–15]. We found evidence of site fidelity (Fig. 2b, c, S4-S24), with individuals returning to the same foraging areas across multiple nights/weeks. Site fidelity has also been observed in other nocturnal species, including flying foxes and some bat species, and is a potential mechanism to increase predictability of foraging success and reduce intraspecific competition [23, 48].

Similar to other central-place foragers [22, 47, 49], including the aforementioned bats [23, 24], oilbirds performed costly commutes from the roost to their foraging areas via non-stop flights, even to distant sites (Fig. 3a, also see [28, 50]), instead of searching for food near the roost. This suggests a benefit of having knowledge of the environment, which enables individuals to track resources, particularly the asynchronous ripening of fruit [51, 52]. The exploratory and excursion patterns we identified (Fig. 1c, f) could facilitate this spatial information acquisition by assessing potential future foraging sites, similar to the behaviour reported for nomadic species such as snail kites [53] and Pacific black ducks [54]. The ability to collect information on fruiting phenology and track habitat-wide resource availability allows individuals to relocate to alternative foraging sites as local resources become depleted or when more profitable patches become available.

Individuals that adopted an ITM strategy exhibited overall lower movement effort over the sampling period compared to roost-faithful and venturer individuals. This counter-intuitive finding can be explained by the ITM prolonged roosting near foraging areas, which reduced the frequency of daily long commutes. This aligns with observations, for example, on the common blackbird, a partially migratory species, where migratory and resident individuals have similar movement effort [55]. The reduced movement effort of intra-tropical migratory oilbirds during the sampling period suggests that the cost of migration flights is offset by increased access to profitable foraging sites. These savings during the non-breeding period may improve body condition at the onset of the breeding season, as observed in grey-headed albatrosses [21], with potential fitness consequences [6, 15]. However, these apparent advantages likely involve trade-offs such as increased predation risk at temporary roosts, compared to more costly strategies (i.e. venturers and roost-faithful). Moreover, strategies that require more effort may confer offsetting benefits, as observed in lesser kestrels where individuals with higher energy expenditure also have greater reproductive success [6]. Our data are insufficient to assess whether these strategies persist over longer periods or whether they affect fitness or survival. For example, we could expect that, despite the higher effort observed in the non-breeding period, individuals foraging near the cave might have an advantage during chick rearing, a period of exceptionally high energetic strain not covered by our sampling period. Moreover, individuals could switch to a different strategy in different years or seasons, possibly depending on the variation on resource availability and distribution, as has been observed in tropical nectarivores [56, 57]. Individuals in our study exhibited moderate repeatability in total nightly flight time, which is within the range of estimates reported for behavioural traits [58] and spatial use in wild animals [59]. This could be related to the observed site fidelity, and repeated visits to foraging sites, while the amount of unexplained variance suggests individual plasticity, allowing oilbirds to respond to changes in resource availability, weather and their own internal state.

## Conclusions

We show that individual variation in movement strategies within a colonial obligate frugivore population resulted in marked differences in movement effort and spatial distribution, with implications that challenge expectations for central-place foragers and optimal foraging. ITM individuals exhibited lower overall movement effort compared to roost-faithful and venturer strategies, despite the cost of long-distance movements, by using temporary roosts near the foraging sites that reduce the frequency of daily commutes. This pattern aligns with findings in blackbirds [55] and kittiwakes [15], suggesting that spatial flexibility can offset movement effort. Our interpretation of movement effort as being related to energetic cost is necessarily cautious and should be viewed as a proxy rather than a direct estimate of energy expenditure. Studies using higher-resolution accelerometry show that DBA (and derivatives thereof) can correlate closely with daily energy expenditure, and that flapping activity often covaries with distance over commuting trips [37–41]. Given our coarser, single-axis ACC data and the possibility of behaviours (e.g. hovering or manoeuvring) that may add effort without contributing much to distance covered, we treat total nightly flight time as an index of relative movement effort that may covary with energetic cost, while deliberately avoiding quantitative claims about absolute energy expenditure. Advances in tracking technology may eventually allow more precise and accurate quantification of energy expenditure and movement strategies in this system. Regardless of the movement strategy, oilbirds showed site fidelity to multiple foraging areas, suggesting knowledge of resource distribution and the ability to track fruit availability across a large area. Our findings reveal the behavioural flexibility of oilbirds and give insights into how alternative movement strategies facilitate coexistence within colonial populations. This further highlights the role of behavioural flexibility in allowing colonial species to adapt to a changing environment while balancing energetic trade-offs. Future studies should explore the long-term persistence of these movement strategies and evaluate how individual variation in movement cost influences fitness, resilience to habitat loss, and the maintenance of ecosystem services such as seed dispersal and habitat connectivity, given the exceptional movement capabilities of oilbirds and their importance as long-distance dispersers [60].

## Supplementary Information

Below is the link to the electronic supplementary material.

Supplementary Material 1

## Data Availability

Data available on the Movebank repository: Holland RA, Wikelski M, Kuemmeth F, Bosque C. 2012. Data from: The secret life of oilbirds: new insights into the movement ecology of a unique avian frugivore. Movebank Data Repository 10.5441/001/1.35fs26kq

## Declarations

### Declaration of AI use

We have not used AI-assisted technologies in creating this article.

## Abbreviations

ACC: Accelerometer
CI: Confidence interval
DBA: Dynamic body acceleration
GPS: Global Positioning System
ITM: Intra-tropical migratory, LMM: Linear mixed-effects model
ODBA: Overall dynamic body acceleration
REML: Restricted maximum likelihood
SD: Standard deviation
VeDBA: Vectorial dynamic body acceleration
