# Supplementary material for: Oilbirds performing non-seasonal intra-tropical migrations reduce flight effort compared to individuals that frequently return to the colony roost
Source: Mov Ecol. 2026 Jul 28;14:53. doi: 10.1186/s40462-026-00686-2 (PMC13418825; doi:10.1186/s40462-026-00686-2)
Supplement: Supplementary file 1 — Supplementary Material 1 [file 40462_2026_686_MOESM1_ESM.docx]

**Oilbirds performing intra-tropical migrations reduce flight effort compared to individuals that frequently return to the colony roost**

Gustavo Alarcón-Nieto*^1,2^, Adriana A. Maldonado-Chaparro^1,3^, Carlos Bosque^4^, Franz Kümmeth^5^, Kamran Safi^1,2^, Martin Wikelski^1,2^

1 Department of Migration, Max Planck Institute of Animal Behavior, 78315 Radolfzell, Germany.

2 Department of Biology, University of Konstanz, Universitätsstraße 10, 78464 Konstanz, Germany.

3 Department of Biology, Universidad del Rosario, Bogotá D.C., Colombia.

4 Departamento de Biología de Organismos, Universidad Simón Bolívar, Caracas, Venezuela.

5 e-obs GmbH, Grünwald, Germany.

*Corresponding author: galarconnieto@ab.mpg.de

**SUPPLEMENTARY MATERIALS**

**Table S1.** Summary of data per individual. Revisited areas are foraging sites visited repeatedly within a 10 km radius. Outside roosting refers to the proportion of the total ACC nights spent roosting outside, determined using ACC data and length of the next night commute. Outside roost is considered if the outbound commute is equal or shorter than the individual's mean foraging flight duration. The distribution of foraging areas includes data on location and number of visits to each area. Visits are at least 1 week apart due to data collection regime. ITM refers to intra-tropical migratory (See main text for details). * The reported distances are measured from the tagging site and do not necessarily represent daily commuting distances.

| **Id** | **GPS nights** | **ACC nights** | **Movement strategy** | **total nightly flight time mean(SD)** | **Outbound commute mean(SD)** | **Outside roosting** | **Foraging areas revisited** | **Distribution of foraging areas distance * (bearing) x revisits** |
| --- | --- | --- | --- | --- | --- | --- | --- | --- |
| #79919 | 8 | 75 | Roost-faithful | 3.44h(1.03) | 1.01h(0.57) | 0.05 | 3 | 75km(114°)x2, 58km(108°)x4, 54km (99°)x5 |
| #79935 | 9 | 102 | Roost-faithful | 3.16h(1.18) | 0.92h(0.67) | 0.05 | 2 | 72km (90°)x4, 7km (33°)x3 |
| #79905 | 7 | 65 | Venturer | 2.66h(0.8) | 0.87h(0.53) | 0.02 | 1 | 37km (100°)x6 |
| #79909 | 11 | 125 | Venturer | 3.2h(0.93) | 1.2h(0.8) | 0.12 | 3 | 33km (-93°)x3, 7km (33°)x2, 54km (77°)x6 |
| #79911 | 8 | 43 | Venturer | 2.39h(1) | 0.56h(0.44) | 0.05 | 2 | 72km (90°)x3, 7km (33°)x2 |
| #79913 | 15 | 140 | Venturer | 3.36h(1.3) | 1.19h(0.71) | 0.09 | 5 | 33km (-93°)x2, 35km (-90°)x2, 43km (89°)x2, 72km (90°)x9, 6km (-2°)x2 |
| #79915 | 14 | 135 | Venturer | 3.81h(1.46) | 1.3h(0.88) | 0.12 | 2 | 68km (92°)x7, 72km (90°)x6 |
| #79917 | 8 | 77 | Venturer | 2.77h(1.69) | 1.04h(1.32) | 0.19 | 1 | 106km (122°)x5 |
| #79921 | 13 | 103 | Venturer | 3.69h(0.75) | 1.53h(0.6) | 0.04 | 2 | 60km (112°)x9, 58km (108°)x5 |
| #79923 | 13 | 33 | Venturer | 2.54h(0.99) | 1.06h(0.59) | 0.09 | 2 | 28km (-93°)x4, 72km (90°)x8 |
| #79927 | 9 | 110 | Venturer | 2.76h(0.99) | 1.15h(0.61) | 0.01 | 2 | 44km (103°)x5, 5km (-76°)x3 |
| #79931 | 15 | 137 | Venturer | 3.2h(0.92) | 1.07h(0.6) | 0.09 | 3 | 58km (108°)x10, 34km (103°)x5, 56km (76°)x2 |
| #79933 | 14 | 119 | Venturer | 2.06h(1.07) | 0.66h(0.56) | 0.06 | 3 | 34km (103°)x10, 2km (20°)x4, 72km (90°)x2 |
| #79972 | 6 | 57 | Venturer | 1.96h(1.14) | 0.57h(0.51) | 0.11 | 1 | 58km (108°)x2 |
| #80007 | 3 | 31 | Venturer | 2.7h(0.71) | 0.9h(0.26) | 0.06 | 2 | 26km (-97°)x2, 35km (-90°)x2 |
| #80198 | 18 | 176 | Venturer | 3.29h(1.14) | 1.04h(0.83) | 0.25 | 3 | 121km (120°)x2, 35km (-90°)x3, 72km (90°)x11 |
| #79907 | 12 | 128 | ITM | 2.61h(1.19) | 0.54h(0.58) | 0.39 | 4 | 33km (-93°)x3, 2km (20°)x2, 72km (90°)x4, 54km (77°)x3 |
| #79929 | 17 | 140 | ITM | 2.39(1.49) | 0.66(0.74) | 0.26 | 3 | 297 km (112°) x8, 60 km (112°) x5, 5 km (-76°) x3 |
| #80009 | 14 | 135 | ITM | 2.98(1.13) | 0.84(0.55) | 0.12 | 4 | 60 km (112°) x5, 33 km (-93°) x4, 134 km (65°) x2, 146 km (67°) x4 |
| #80015 | 6 | 64 | ITM | 2.03(0.79) | 0.48(0.42) | 0.28 | 3 | 44 km (103°) x2, 2 km (20°) x2, 54 km (77°) x2 |
| #80017 | 18 | 132 | ITM | 1.2(1.02) | 0.29(0.41) | 0.42 | 3 | 6 km (-2°) x3, 113 km (56°) x8, 112 km (55°) x3 |

**Table S2**. Minimum (min_dist_m) and median (median_dist_m) distance for each pair of individuals (Ind A – Ind B) during the entire sampling period, based on all the locations collected after the end of commute-oy flights and the start of the commute-in flight each night. These locations were classified as foraging sites. Note that the mean GPS error was 12m (SD = 10m) and that all locations were included, regardless of time, so short distances do not necessarily represent birds coinciding in time and space.

| **Ind A** | **Ind B** | **min_dist_m** | **median_dist_m** |
| --- | --- | --- | --- |
| X.80009 | X.79923 | 2.272 | 78671.917 |
| X.79907 | X.79913 | 9.107 | 42673.120 |
| X.79935 | X.79923 | 18.352 | 45165.364 |
| X.79917 | X.79905 | 25.037 | 73626.320 |
| X.79935 | X.80009 | 52.408 | 61119.831 |
| X.79927 | X.80015 | 60.767 | 28304.700 |
| X.80011 | X.79923 | 65.948 | 76296.923 |
| X.79935 | X.80011 | 75.865 | 36390.118 |
| X.80198 | X.80009 | 76.552 | 78373.220 |
| X.79935 | X.80017 | 76.812 | 70790.741 |
| X.79907 | X.79909 | 89.132 | 43555.855 |
| X.79929 | X.79931 | 92.488 | 63163.830 |
| X.80198 | X.79923 | 96.459 | 41646.684 |
| X.79909 | X.79970 | 221.223 | 27914.714 |
| X.80017 | X.79923 | 247.807 | 67591.838 |
| X.79931 | X.79921 | 257.468 | 24783.266 |
| X.79929 | X.80009 | 271.112 | 129913.715 |
| X.79927 | X.79923 | 272.458 | 31291.270 |
| X.79935 | X.79913 | 276.389 | 52112.528 |
| X.79933 | X.79972 | 294.592 | 42357.646 |
| X.79919 | X.79921 | 349.426 | 12853.532 |
| X.79935 | X.79909 | 369.194 | 44048.562 |
| X.79931 | X.80013 | 373.368 | 24930.305 |
| X.79909 | X.79913 | 395.845 | 38542.647 |
| X.79933 | X.80015 | 410.285 | 29564.867 |
| X.79935 | X.79907 | 432.553 | 41420.654 |
| X.79909 | X.80017 | 444.497 | 64894.830 |
| X.79915 | X.79911 | 470.238 | 36876.926 |
| X.79933 | X.79923 | 485.319 | 38629.295 |
| X.80015 | X.79923 | 510.238 | 40546.435 |
| X.80198 | X.79935 | 523.581 | 68088.970 |
| X.79935 | X.79911 | 525.545 | 46839.654 |
| X.80011 | X.79972 | 526.895 | 85086.382 |
| X.79907 | X.79923 | 528.670 | 41382.845 |
| X.80198 | X.79907 | 531.998 | 69495.468 |
| X.79931 | X.79919 | 546.103 | 21503.058 |
| X.79907 | X.79911 | 551.113 | 42052.952 |
| X.79913 | X.79923 | 567.074 | 30975.337 |
| X.79907 | X.79915 | 603.887 | 50192.130 |
| X.80011 | X.79905 | 606.539 | 68361.324 |
| X.80198 | X.79913 | 621.369 | 35011.788 |
| X.79909 | X.79923 | 625.611 | 35684.973 |
| X.80198 | X.80011 | 642.310 | 103318.343 |
| X.79933 | X.79905 | 643.903 | 20356.770 |
| X.79909 | X.79915 | 649.627 | 38787.872 |
| X.79909 | X.79911 | 681.661 | 38653.799 |
| X.79927 | X.79929 | 683.033 | 67318.090 |
| X.79931 | X.80015 | 688.335 | 31095.505 |
| X.79911 | X.79913 | 697.033 | 40206.134 |
| X.80011 | X.80009 | 697.239 | 91529.312 |
| X.79909 | X.80009 | 701.305 | 69320.953 |
| X.79933 | X.79921 | 702.933 | 26144.659 |
| X.79929 | X.79919 | 724.248 | 69908.144 |
| X.80015 | X.79972 | 726.691 | 52907.255 |
| X.79933 | X.79919 | 741.744 | 24212.861 |
| X.79915 | X.79913 | 753.463 | 18734.241 |
| X.80198 | X.79909 | 753.705 | 50368.497 |
| X.79907 | X.80017 | 754.532 | 68821.119 |
| X.79915 | X.79931 | 774.961 | 26980.627 |
| X.80198 | X.80007 | 782.415 | 98437.867 |
| X.79933 | X.79911 | 813.222 | 40042.317 |
| X.79933 | X.79909 | 813.554 | 34420.278 |
| X.80011 | X.79970 | 818.925 | 87645.199 |
| X.79909 | X.80007 | 841.882 | 80909.399 |
| X.79933 | X.79907 | 887.370 | 36593.847 |
| X.79929 | X.79909 | 899.913 | 90243.337 |
| X.79929 | X.79921 | 904.126 | 63970.202 |
| X.80007 | X.79923 | 911.126 | 90371.448 |
| X.80007 | X.80009 | 928.604 | 89996.755 |
| X.79935 | X.80007 | 941.768 | 36541.129 |
| X.79905 | X.79972 | 945.634 | 41690.453 |
| X.79970 | X.79905 | 955.289 | 32476.376 |
| X.79911 | X.79923 | 967.106 | 43923.696 |
| X.80017 | X.80009 | 974.995 | 91218.592 |
| X.79929 | X.79935 | 988.400 | 88469.519 |
| X.80011 | X.80007 | 1017.147 | 5245.382 |
| X.79917 | X.79913 | 1046.218 | 60555.613 |
| X.79933 | X.79931 | 1077.995 | 24014.318 |
| X.79929 | X.80017 | 1079.797 | 116317.707 |
| X.79972 | X.79921 | 1152.690 | 53767.476 |
| X.79929 | X.79913 | 1158.571 | 91544.534 |
| X.79929 | X.79917 | 1182.725 | 110427.540 |
| X.79933 | X.79927 | 1208.541 | 20700.932 |
| X.79907 | X.80011 | 1214.288 | 40028.052 |
| X.79927 | X.79919 | 1236.621 | 15205.255 |
| X.79933 | X.80011 | 1281.995 | 66601.302 |
| X.79970 | X.80015 | 1298.986 | 32736.907 |
| X.79915 | X.80015 | 1306.331 | 41528.304 |
| X.79929 | X.79923 | 1327.513 | 88398.762 |
| X.79927 | X.79972 | 1335.807 | 43313.451 |
| X.79970 | X.79972 | 1345.572 | 54433.247 |
| X.79909 | X.79931 | 1351.546 | 31321.577 |
| X.80017 | X.80015 | 1356.689 | 80872.001 |
| X.79917 | X.80009 | 1371.411 | 94225.989 |
| X.80198 | X.80013 | 1374.002 | 34299.065 |
| X.80013 | X.79919 | 1379.395 | 11311.768 |
| X.80198 | X.79911 | 1382.680 | 49688.622 |
| X.79931 | X.79913 | 1387.735 | 34824.733 |
| X.79913 | X.80009 | 1429.406 | 74089.514 |
| X.79929 | X.80013 | 1435.424 | 61101.197 |
| X.79919 | X.79972 | 1466.308 | 46019.449 |
| X.79909 | X.80015 | 1467.677 | 35211.278 |
| X.79911 | X.79970 | 1493.300 | 30857.056 |
| X.79935 | X.79915 | 1510.409 | 64997.183 |
| X.79915 | X.79923 | 1530.714 | 25413.696 |
| X.79907 | X.80015 | 1556.112 | 39589.242 |
| X.79931 | X.79972 | 1575.281 | 45777.814 |
| X.79905 | X.80015 | 1603.950 | 26246.604 |
| X.79915 | X.80009 | 1629.834 | 71701.233 |
| X.79927 | X.79921 | 1632.643 | 18092.436 |
| X.79927 | X.80013 | 1643.681 | 13463.512 |
| X.79933 | X.80017 | 1648.973 | 91186.404 |
| X.79935 | X.79970 | 1657.383 | 51349.678 |
| X.80011 | X.79909 | 1692.228 | 51407.932 |
| X.79927 | X.80198 | 1692.290 | 30303.481 |
| X.79933 | X.79913 | 1711.248 | 39378.012 |
| X.79933 | X.79935 | 1723.435 | 38527.747 |
| X.79907 | X.80009 | 1725.593 | 76953.009 |
| X.79933 | X.79915 | 1729.062 | 34279.064 |
| X.80013 | X.79923 | 1729.370 | 34690.639 |
| X.79933 | X.80198 | 1738.346 | 37994.900 |
| X.79909 | X.79972 | 1743.218 | 52767.590 |
| X.79909 | X.79905 | 1744.975 | 30068.473 |
| X.79933 | X.79970 | 1745.222 | 36540.954 |
| X.80011 | X.80015 | 1783.196 | 53207.696 |
| X.79929 | X.79907 | 1802.308 | 91181.264 |
| X.79917 | X.79970 | 1813.548 | 86182.718 |
| X.79931 | X.79970 | 1824.138 | 38693.905 |
| X.79907 | X.79970 | 1834.217 | 44766.667 |
| X.79927 | X.79917 | 1862.905 | 65547.483 |
| X.80198 | X.79970 | 1886.166 | 32184.988 |
| X.80198 | X.80017 | 1912.830 | 68371.702 |
| X.79907 | X.79972 | 1915.190 | 60086.381 |
| X.79911 | X.80017 | 1929.669 | 65974.216 |
| X.79911 | X.80015 | 1938.696 | 35010.758 |
| X.79935 | X.79905 | 1952.178 | 36283.511 |
| X.79913 | X.80017 | 1962.205 | 66174.308 |
| X.79907 | X.80007 | 1963.512 | 34721.920 |
| X.80011 | X.79911 | 1985.558 | 66040.365 |
| X.79915 | X.79970 | 1988.379 | 28248.871 |
| X.79935 | X.80015 | 2014.344 | 40177.282 |
| X.80198 | X.79905 | 2027.547 | 35814.723 |
| X.79933 | X.80013 | 2047.636 | 22224.730 |
| X.80198 | X.79915 | 2051.459 | 23010.042 |
| X.79911 | X.79931 | 2057.131 | 41116.035 |
| X.80013 | X.79921 | 2061.178 | 5710.211 |
| X.80007 | X.79913 | 2067.506 | 99560.431 |
| X.79909 | X.79917 | 2072.529 | 78731.898 |
| X.79911 | X.79972 | 2092.378 | 55612.153 |
| X.79935 | X.79972 | 2116.286 | 55925.973 |
| X.79933 | X.79929 | 2121.906 | 58516.092 |
| X.80013 | X.79905 | 2158.003 | 21643.700 |
| X.79935 | X.79917 | 2189.034 | 68627.818 |
| X.79931 | X.80017 | 2189.984 | 89148.559 |
| X.79911 | X.79905 | 2266.713 | 37718.217 |
| X.79927 | X.79913 | 2269.826 | 31433.852 |
| X.79917 | X.80017 | 2280.539 | 113865.650 |
| X.80013 | X.80009 | 2295.909 | 62490.804 |
| X.80198 | X.79972 | 2308.461 | 61024.176 |
| X.79917 | X.79931 | 2389.559 | 60868.577 |
| X.80198 | X.79919 | 2407.731 | 24740.202 |
| X.79933 | X.79917 | 2421.727 | 73827.974 |
| X.79927 | X.80009 | 2453.029 | 66215.053 |
| X.79917 | X.80013 | 2462.047 | 52361.371 |
| X.79923 | X.79972 | 2466.006 | 61377.179 |
| X.79927 | X.79931 | 2481.334 | 14977.606 |
| X.80198 | X.79917 | 2484.450 | 59603.650 |
| X.79927 | X.79905 | 2525.795 | 13347.131 |
| X.79917 | X.79923 | 2527.700 | 63740.607 |
| X.80198 | X.80015 | 2540.540 | 44922.215 |
| X.79919 | X.80009 | 2549.522 | 78935.250 |
| X.79933 | X.80009 | 2572.911 | 61427.015 |
| X.79970 | X.80017 | 2581.809 | 55950.969 |
| X.79935 | X.79931 | 2582.837 | 42033.017 |
| X.80009 | X.79921 | 2600.001 | 82531.820 |
| X.79929 | X.80198 | 2621.067 | 91982.137 |
| X.79931 | X.79905 | 2656.542 | 23137.931 |
| X.79931 | X.79923 | 2692.864 | 42001.514 |
| X.80017 | X.79972 | 2697.698 | 92850.210 |
| X.80015 | X.80009 | 2754.865 | 60898.786 |
| X.79929 | X.79972 | 2828.752 | 90100.893 |
| X.79911 | X.80009 | 2871.386 | 62538.257 |
| X.79929 | X.79911 | 2899.750 | 78549.684 |
| X.79915 | X.80017 | 2924.766 | 67473.868 |
| X.79929 | X.80015 | 2948.485 | 59791.996 |
| X.79907 | X.79917 | 2983.870 | 73383.434 |
| X.79970 | X.79923 | 2984.955 | 33984.046 |
| X.80013 | X.79972 | 2987.687 | 47588.535 |
| X.79931 | X.80009 | 3011.436 | 61355.036 |
| X.79907 | X.79905 | 3028.320 | 37534.602 |
| X.79970 | X.79913 | 3060.450 | 28107.844 |
| X.80011 | X.79915 | 3093.549 | 100052.535 |
| X.79907 | X.79931 | 3120.633 | 40058.096 |
| X.79915 | X.79905 | 3137.079 | 32126.908 |
| X.80198 | X.79921 | 3315.229 | 30258.237 |
| X.80011 | X.80017 | 3337.380 | 138041.649 |
| X.80011 | X.79913 | 3339.369 | 84427.012 |
| X.80198 | X.79931 | 3344.004 | 41341.731 |
| X.79905 | X.80009 | 3362.575 | 59369.151 |
| X.79905 | X.79923 | 3471.307 | 36521.749 |
| X.79915 | X.80013 | 3528.004 | 29028.867 |
| X.80009 | X.79972 | 3561.512 | 81241.348 |
| X.79913 | X.80015 | 3613.532 | 41018.973 |
| X.79927 | X.79909 | 3619.716 | 30438.593 |
| X.79905 | X.80017 | 3685.578 | 88801.838 |
| X.79915 | X.79972 | 3964.623 | 51418.397 |
| X.79911 | X.79917 | 4016.408 | 74930.089 |
| X.79927 | X.79935 | 4034.552 | 43045.736 |
| X.79905 | X.79913 | 4098.003 | 37149.815 |
| X.79913 | X.79972 | 4099.928 | 60232.306 |
| X.79927 | X.80017 | 4123.581 | 88213.465 |
| X.79917 | X.80015 | 4125.719 | 77635.453 |
| X.80011 | X.79931 | 4182.430 | 71272.513 |
| X.79970 | X.80009 | 4200.102 | 62383.152 |
| X.79915 | X.79917 | 4211.334 | 60720.120 |
| X.79929 | X.79970 | 4276.421 | 63125.176 |
| X.79929 | X.80011 | 4417.160 | 88976.892 |
| X.79929 | X.79905 | 4670.362 | 56313.932 |
| X.80015 | X.79921 | 4707.133 | 40253.827 |
| X.79927 | X.79907 | 4743.196 | 44528.368 |
| X.80013 | X.80015 | 4771.057 | 35125.648 |
| X.79909 | X.80013 | 4788.732 | 36002.436 |
| X.79923 | X.79921 | 4823.475 | 31229.459 |
| X.79917 | X.79972 | 4932.098 | 55840.467 |
| X.79913 | X.79919 | 5044.377 | 26352.221 |
| X.79915 | X.79919 | 5048.829 | 22251.180 |
| X.80013 | X.79913 | 5067.827 | 28400.020 |
| X.79935 | X.80013 | 5164.022 | 55802.027 |
| X.80013 | X.80017 | 5225.636 | 91045.426 |
| X.79919 | X.79923 | 5334.586 | 28600.284 |
| X.79927 | X.79911 | 5596.261 | 35091.179 |
| X.80011 | X.79917 | 5616.063 | 106702.631 |
| X.79929 | X.79915 | 5801.989 | 77513.379 |
| X.79907 | X.80013 | 5928.270 | 40744.562 |
| X.79917 | X.79919 | 6074.060 | 59094.814 |
| X.79927 | X.80011 | 6485.392 | 75679.543 |
| X.80011 | X.80013 | 6611.836 | 86179.759 |
| X.80013 | X.79970 | 6976.103 | 40731.995 |
| X.79927 | X.79970 | 7082.878 | 35018.686 |
| X.79911 | X.79919 | 7121.090 | 37121.134 |
| X.79911 | X.80013 | 7123.211 | 39930.718 |
| X.80015 | X.79919 | 7486.169 | 36642.260 |
| X.80007 | X.79972 | 7719.760 | 84676.700 |
| X.79927 | X.79915 | 8778.878 | 32174.303 |
| X.79907 | X.79919 | 10506.632 | 51007.737 |
| X.79905 | X.79919 | 11117.781 | 23468.505 |
| X.79917 | X.79921 | 11975.980 | 48086.643 |
| X.79905 | X.79921 | 12214.427 | 25941.732 |
| X.79929 | X.80007 | 12562.576 | 90517.201 |
| X.79970 | X.79919 | 12775.512 | 35919.489 |
| X.79915 | X.79921 | 13486.258 | 26726.325 |
| X.79909 | X.79919 | 13630.852 | 44261.790 |
| X.79913 | X.79921 | 14153.417 | 31979.265 |
| X.79911 | X.79921 | 14999.805 | 43686.009 |
| X.79911 | X.80007 | 15450.657 | 72349.711 |
| X.80017 | X.79919 | 15739.532 | 81389.822 |
| X.79907 | X.79921 | 17146.415 | 56918.297 |
| X.80011 | X.79919 | 18293.638 | 85687.263 |
| X.79935 | X.79919 | 18879.058 | 52487.514 |
| X.79927 | X.80007 | 18998.186 | 70815.672 |
| X.80007 | X.79905 | 19838.853 | 63461.377 |
| X.80017 | X.79921 | 20695.732 | 89730.054 |
| X.79917 | X.80007 | 24150.856 | 128425.985 |
| X.80013 | X.80007 | 24486.299 | 81968.229 |
| X.79935 | X.79921 | 25151.319 | 59015.345 |
| X.80007 | X.80017 | 26381.638 | 136478.066 |
| X.79909 | X.79921 | 27890.784 | 37792.513 |
| X.79933 | X.80007 | 28010.321 | 61556.405 |
| X.80007 | X.80015 | 28308.749 | 60422.382 |
| X.80007 | X.79970 | 29111.762 | 85698.630 |
| X.79970 | X.79921 | 29645.550 | 45835.469 |
| X.79915 | X.80007 | 32640.656 | 100340.142 |
| X.79931 | X.80007 | 34222.372 | 83472.636 |
| X.80011 | X.79921 | 44607.946 | 89082.870 |
| X.80007 | X.79919 | 47655.312 | 86575.712 |
| X.80007 | X.79921 | 73057.584 | 87511.057 |


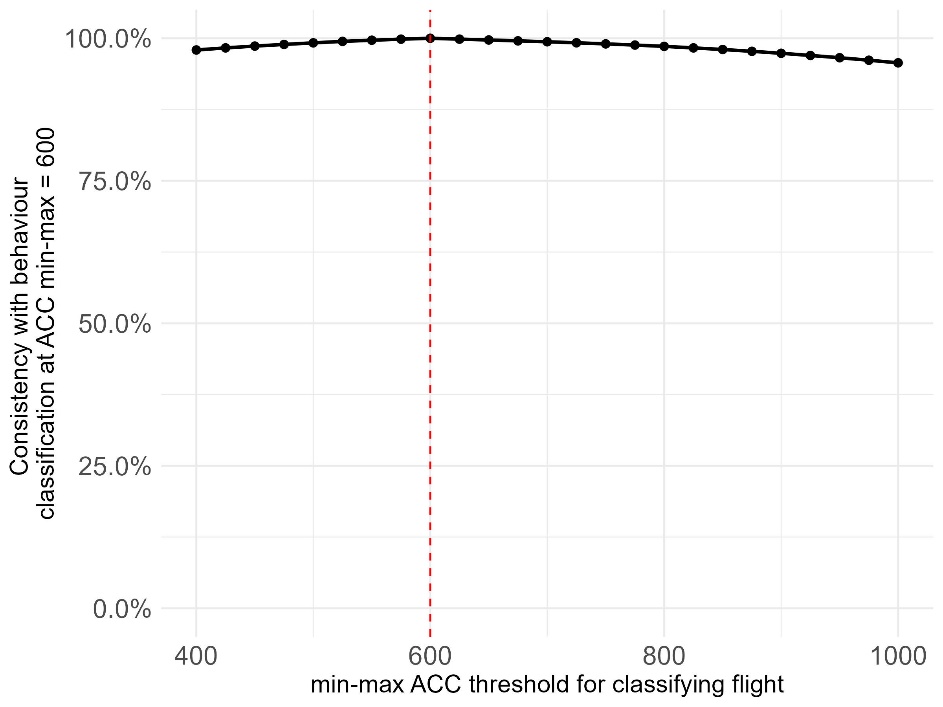


**Figure S1.** Sensitivity analysis for the classification of flight behaviour using single-axis ACC data, based on a min-max value of 600. Values above 600 were classified as flight, as a signal of wing flapping, while lower values were labeled as roosting. This approach expands on previous qualitative observations by Holland et al. [28]


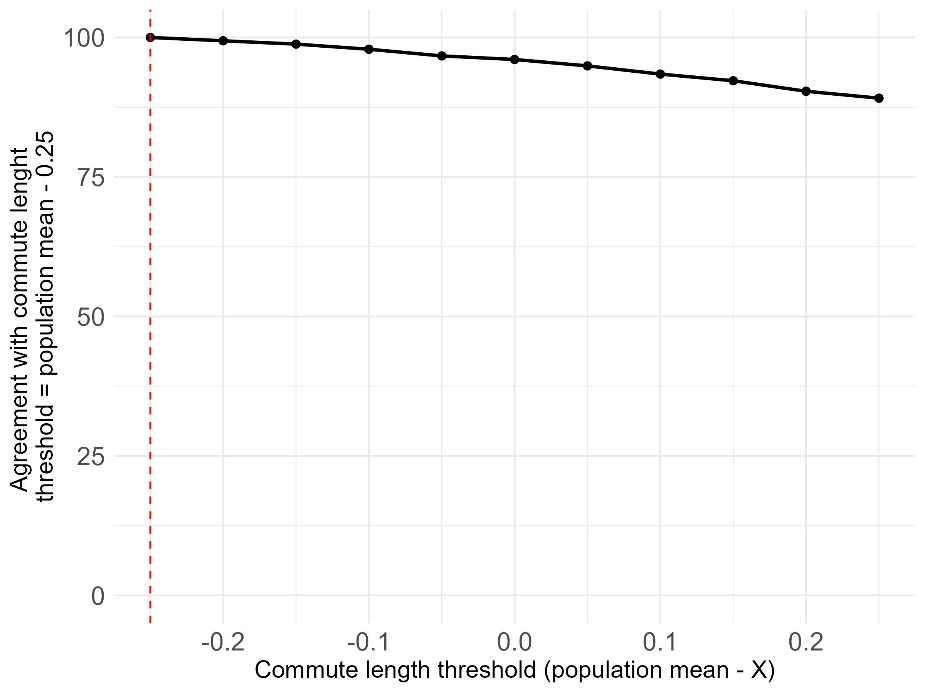


**Figure S2.** Sensitivity analysis for the classification of nocturnal movement patterns, symmetric nights (Round-trips) with long and with short commutes, based the population mean commute time (3865 s, SD = 2496.6 s). In the figure, X represents the threshold used to separate the two categories. In our initial classification, values above mean – 0.25SD (3632 s) were classified as long commutes and below the threshold, short commutes.


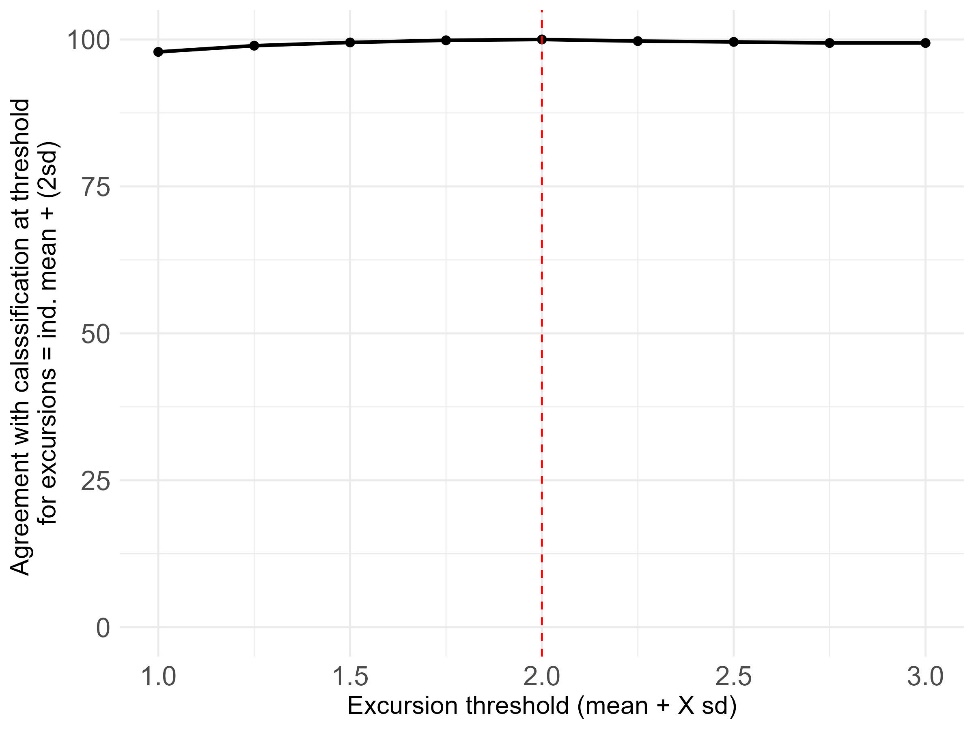


**Figure S3.** Sensitivity analysis for the classification of nocturnal movement pattern excursions, which describe the longest flights of each individual, defined as the mean nightly flight time for the individual + 2SD. In the figure, X represents this modifier.


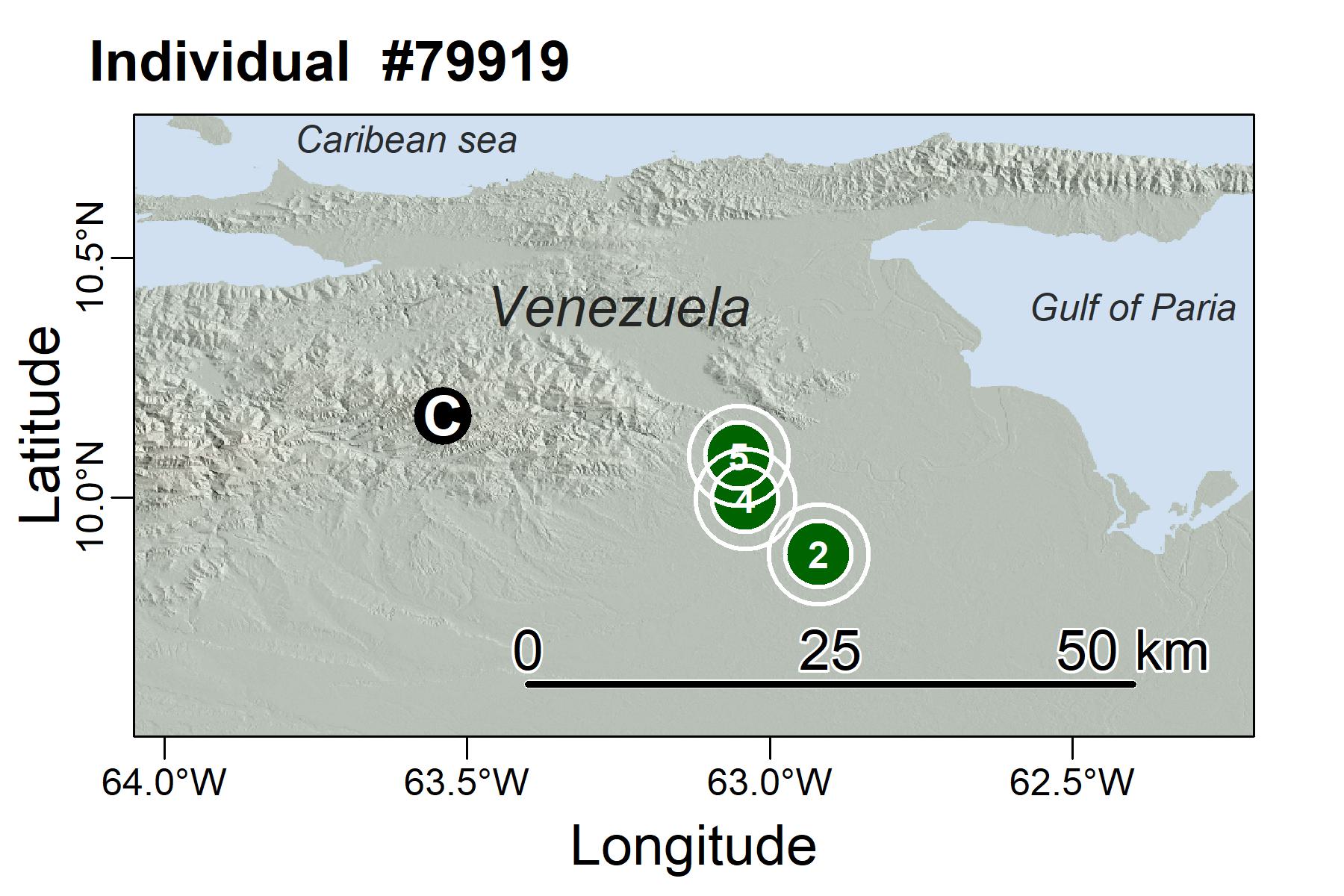


**Figure S4.** Site fidelity single individual to multiple foraging areas during the sampling period (Oilbird #79919, 79 GPS locations over 8 weeks), the numbers in the green circles are nights (revisits) at each site. C in a black circle represents the tagging site, Cueva del guacharo


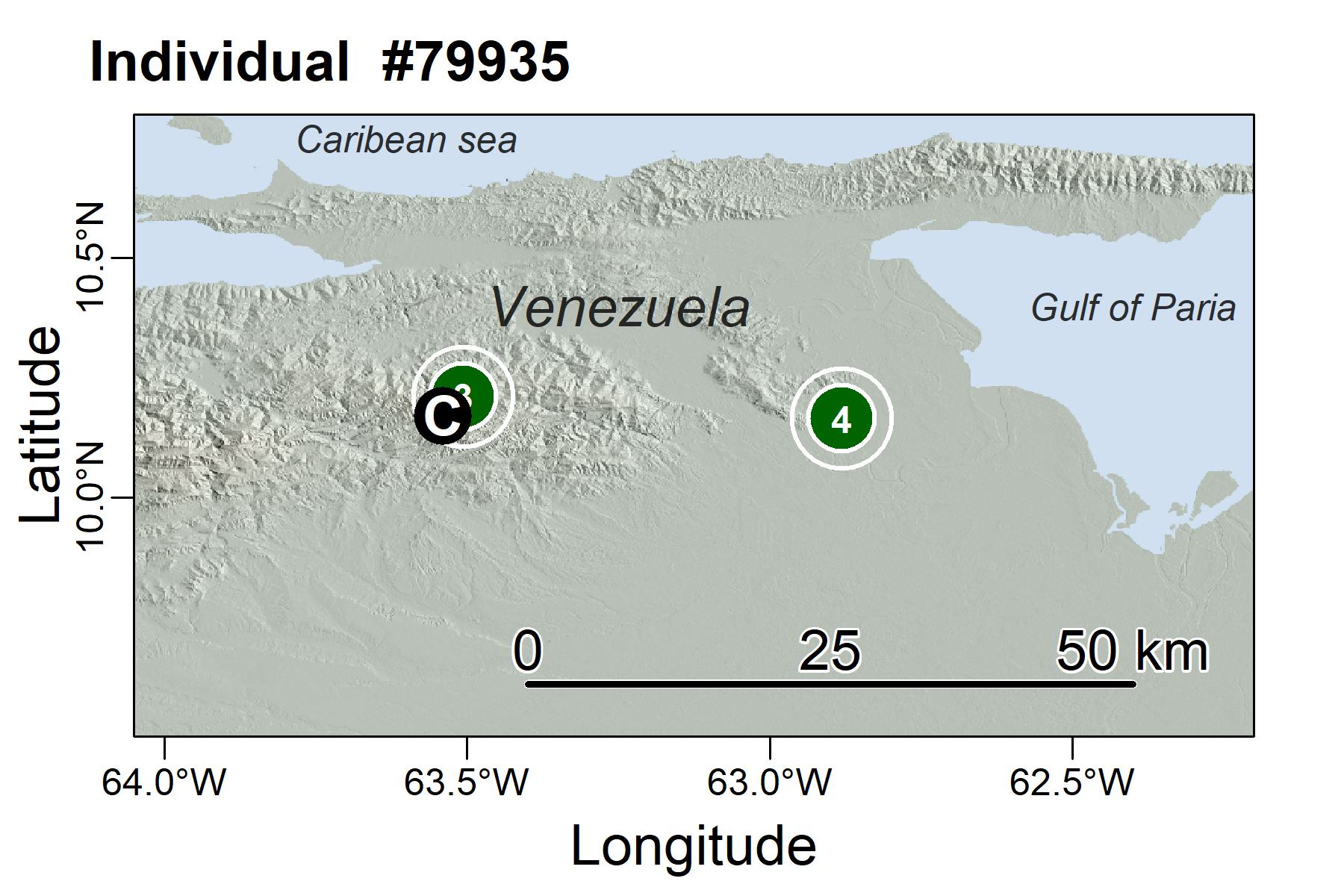


**Figure S5.** Site fidelity single individual to multiple foraging areas during the sampling period (Oilbird #79935, 52 GPS locations over 9 weeks), the numbers in the green circles are nights (revisits) at each site. C in a black circle represents the tagging site, Cueva del guacharo


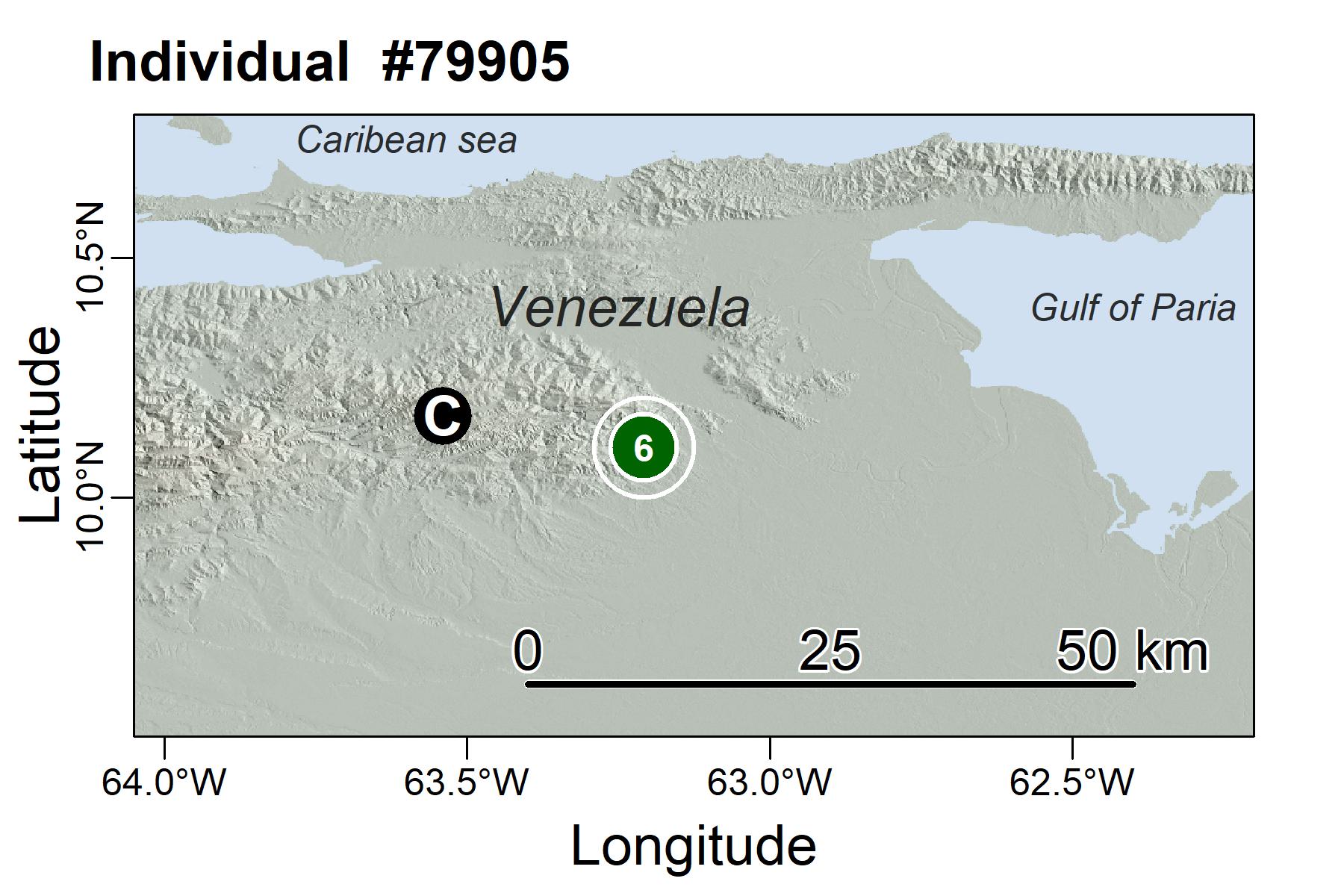


**Figure S6.** Site fidelity single individual to multiple foraging areas during the sampling period (Oilbird #79905, 77 GPS locations over 7 weeks), the numbers in the green circles are nights (revisits) at each site. C in a black circle represents the tagging site, Cueva del guacharo.


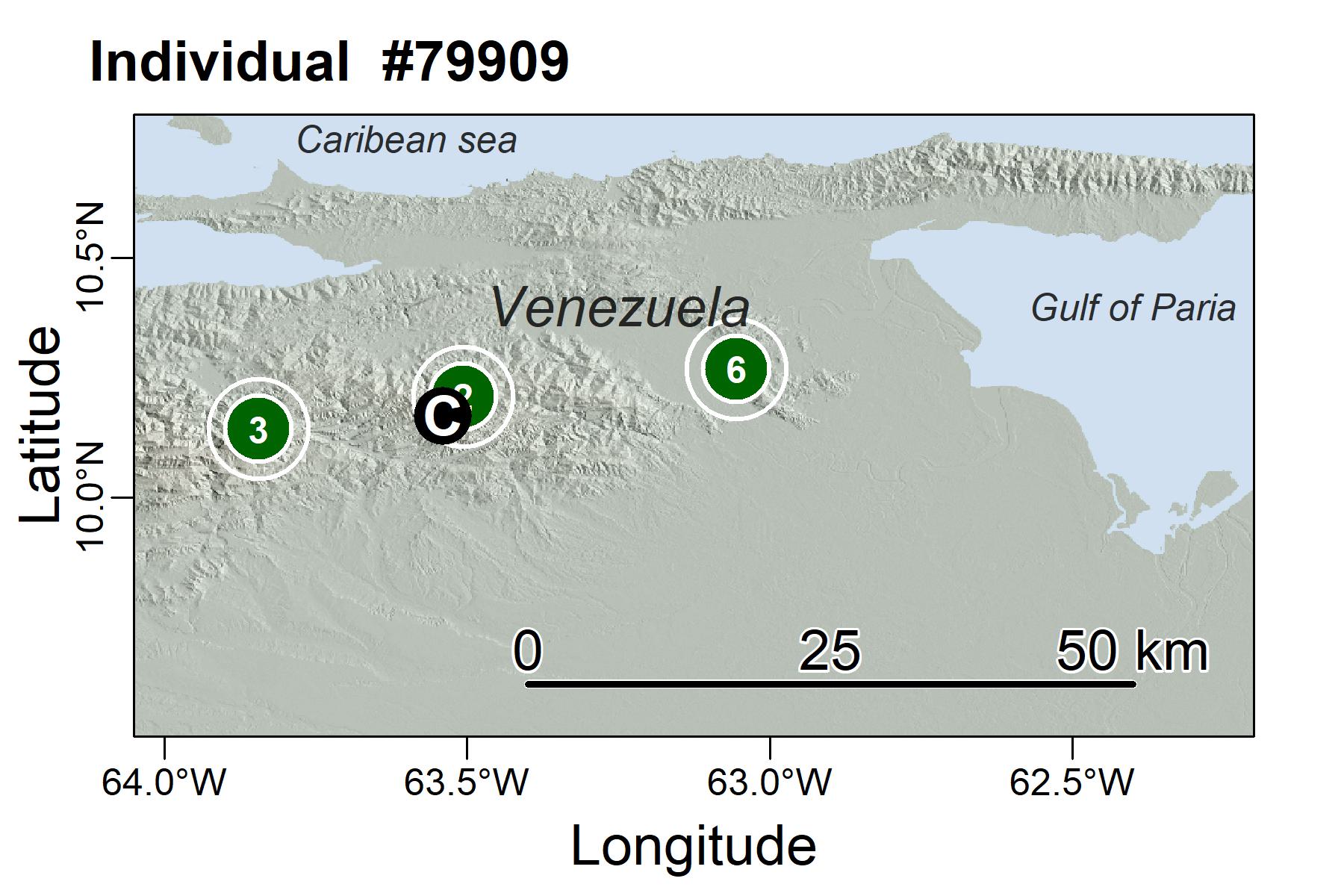


**Figure S7.** Site fidelity single individual to multiple foraging areas during the sampling period (Oilbird #79909, 111 GPS locations over 11 weeks), the numbers in the green circles are nights (revisits) at each site. C in a black circle represents the tagging site, Cueva del guacharo.


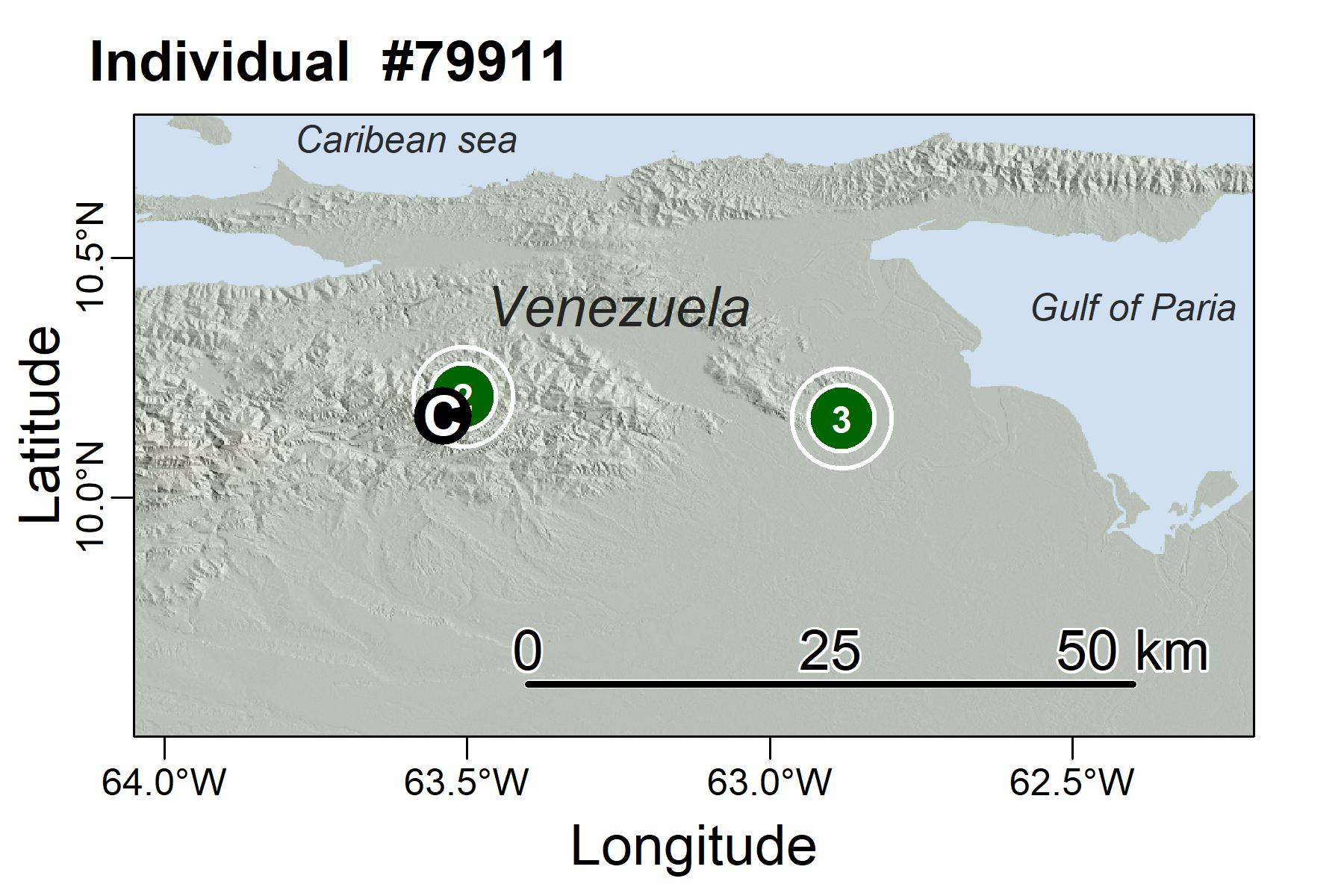


**Figure S8.** Site fidelity single individual to multiple foraging areas during the sampling period (Oilbird #79911, 74 GPS locations over 8 weeks), the numbers in the green circles are nights (revisits) at each site. C in a black circle represents the tagging site, Cueva del guacharo.


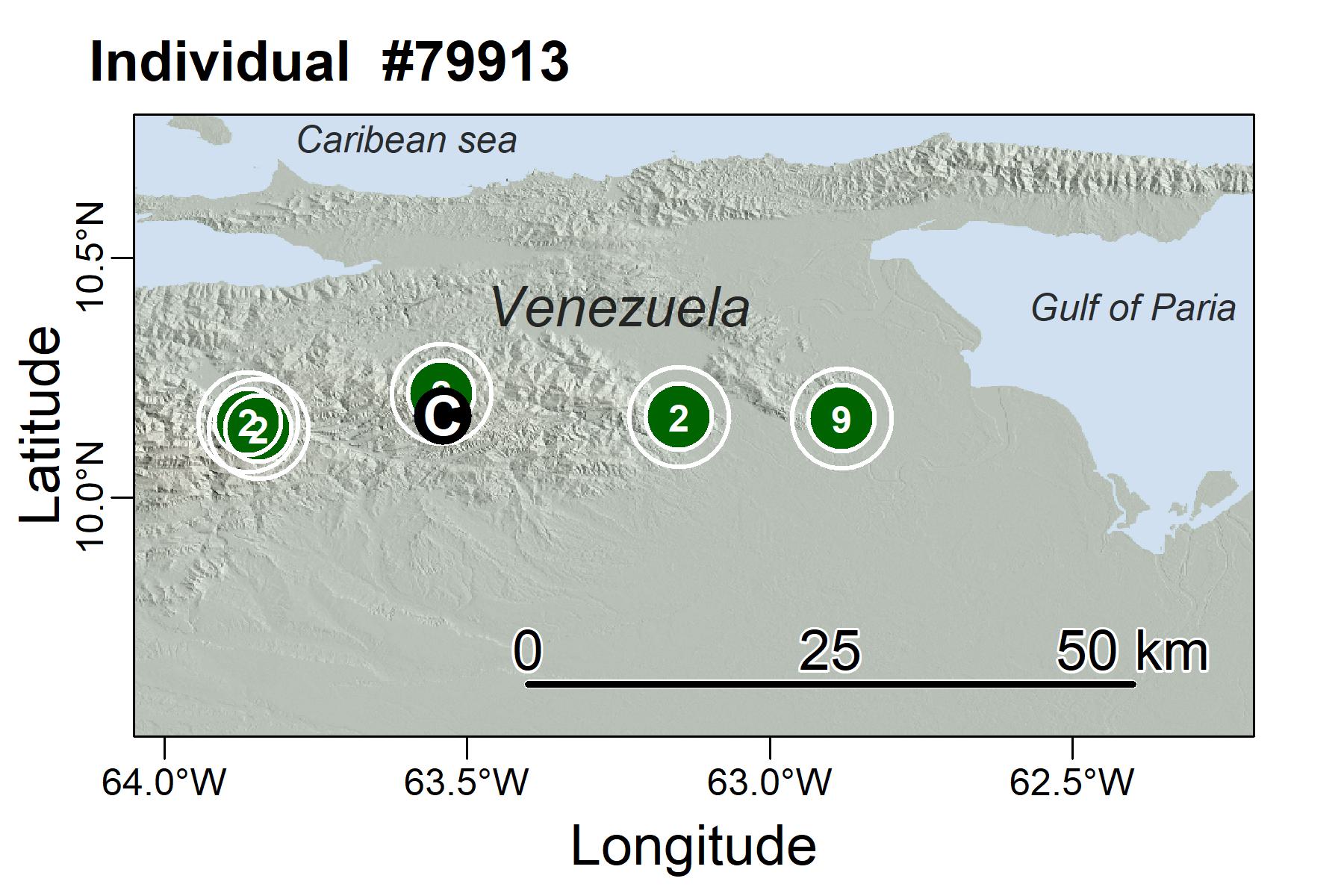


**Figure S9.** Site fidelity single individual to multiple foraging areas during the sampling period (Oilbird #79913, 138 GPS locations over 15 weeks), the numbers in the green circles are nights (revisits) at each site. C in a black circle represents the tagging site, Cueva del guacharo.


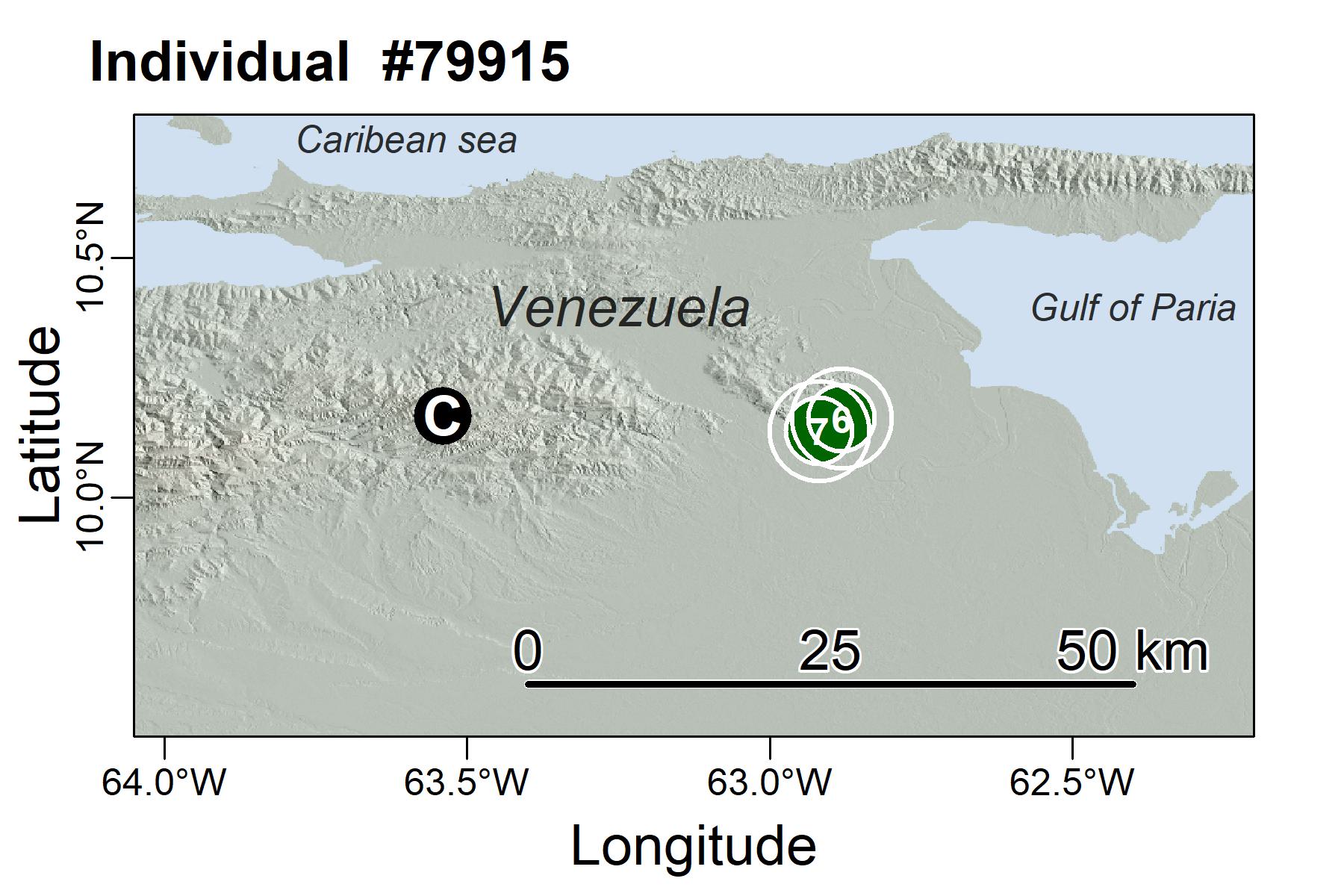


**Figure S10.** Site fidelity single individual to multiple foraging areas during the sampling period (Oilbird #79915, 137 GPS locations over 14 weeks), the numbers in the green circles are nights (revisits) at each site. C in a black circle represents the tagging site, Cueva del guacharo.


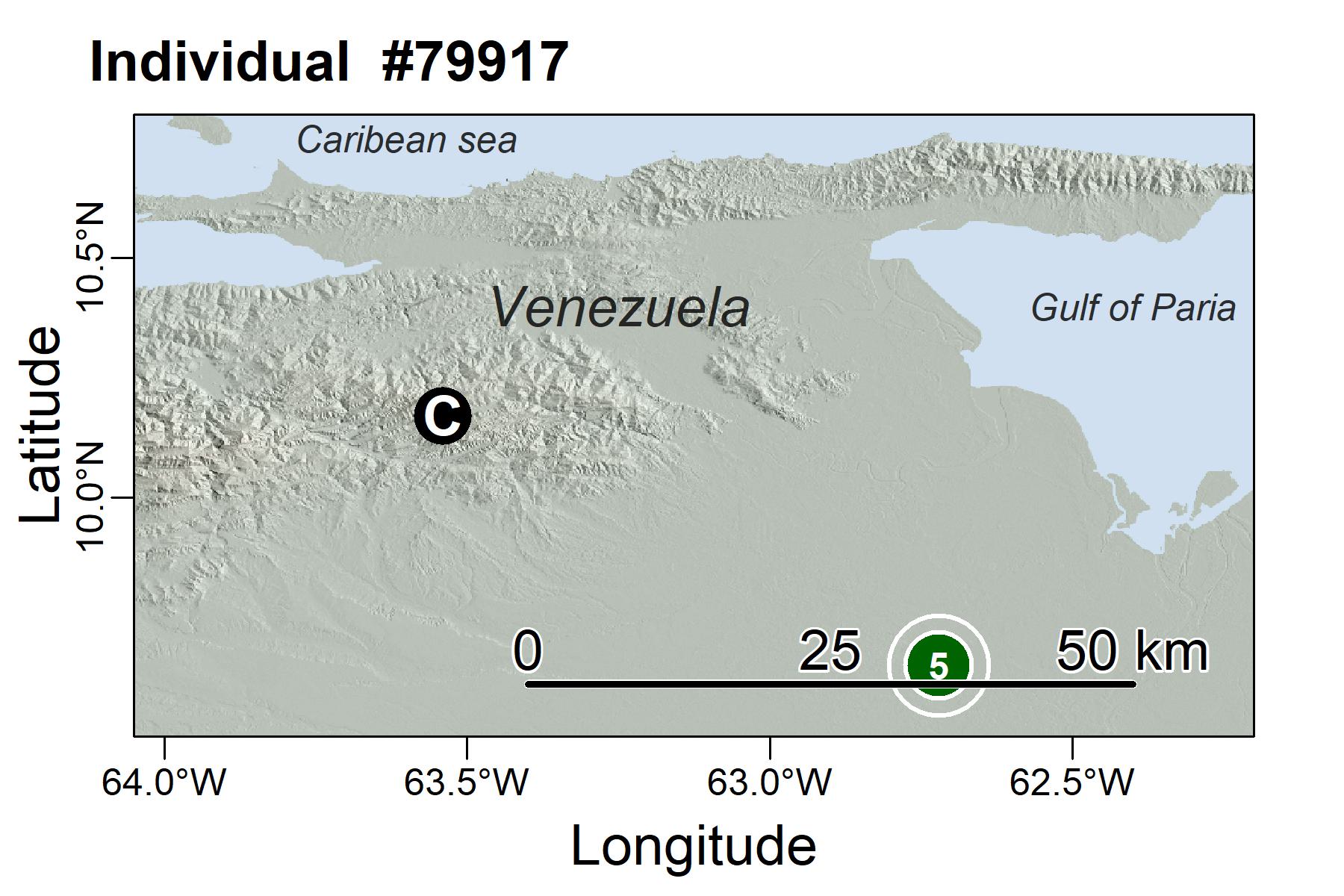


**Figure S11.** Site fidelity single individual to multiple foraging areas during the sampling period (Oilbird #79917, 81 GPS locations over 8 weeks), the numbers in the green circles are nights (revisits) at each site. C in a black circle represents the tagging site, Cueva del guacharo.


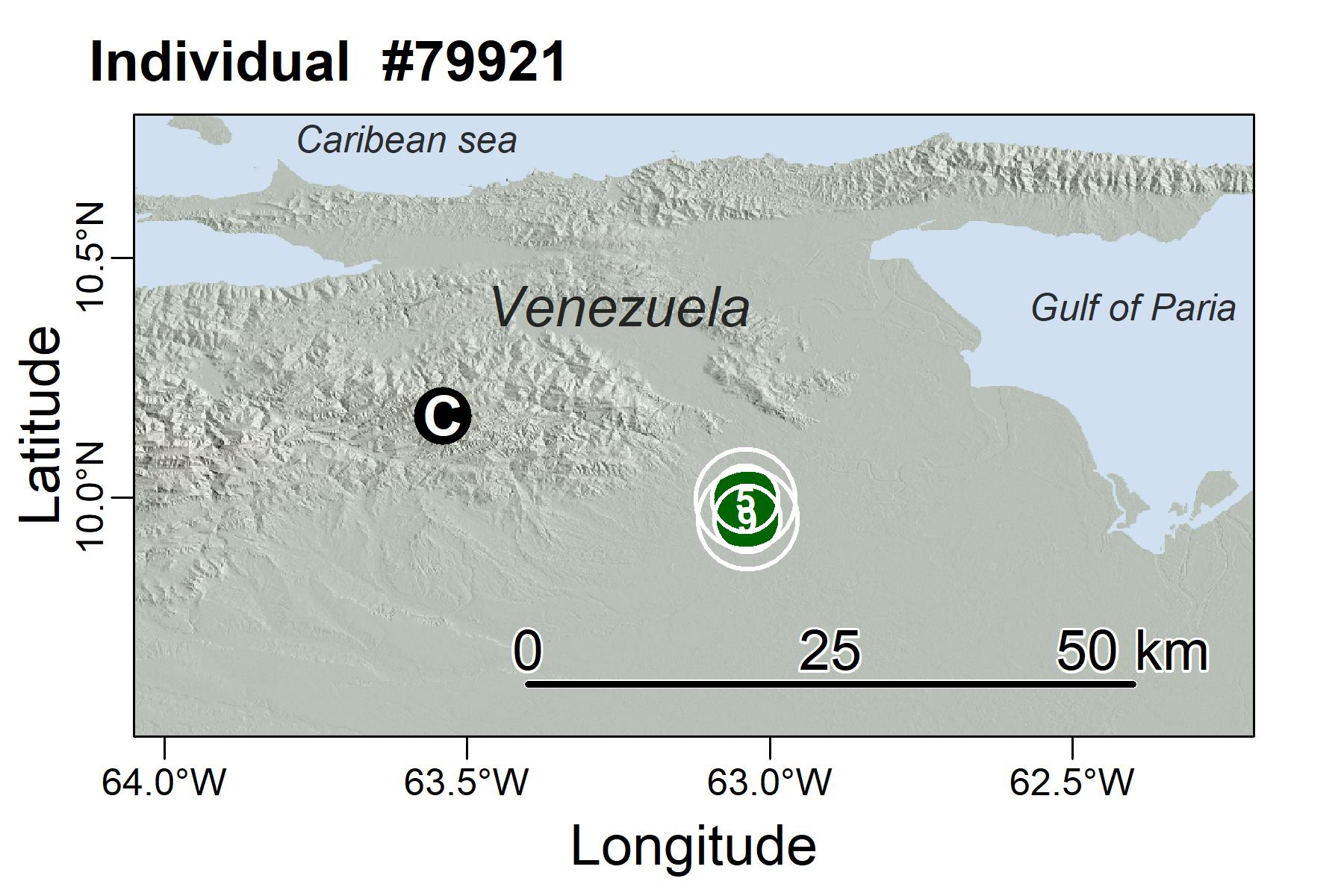


**Figure S12.** Site fidelity single individual to multiple foraging areas during the sampling period (Oilbird #79921, 147 GPS locations over 13 weeks), the numbers in the green circles are nights (revisits) at each site. C in a black circle represents the tagging site, Cueva del guacharo.


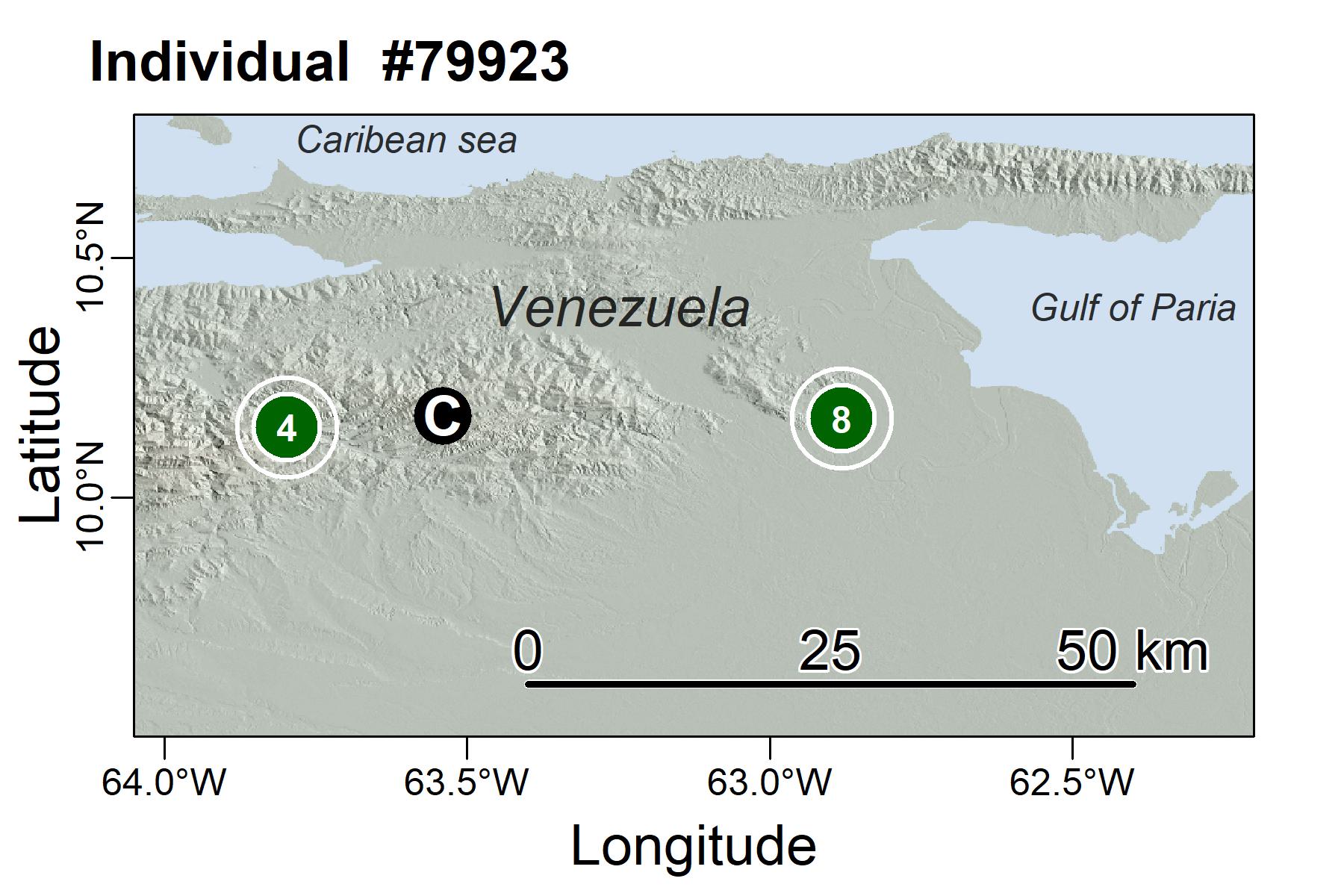


**Figure S13.** Site fidelity single individual to multiple foraging areas during the sampling period (Oilbird #79923, 104 GPS locations over 13 weeks), the numbers in the green circles are nights (revisits) at each site. C in a black circle represents the tagging site, Cueva del guacharo.


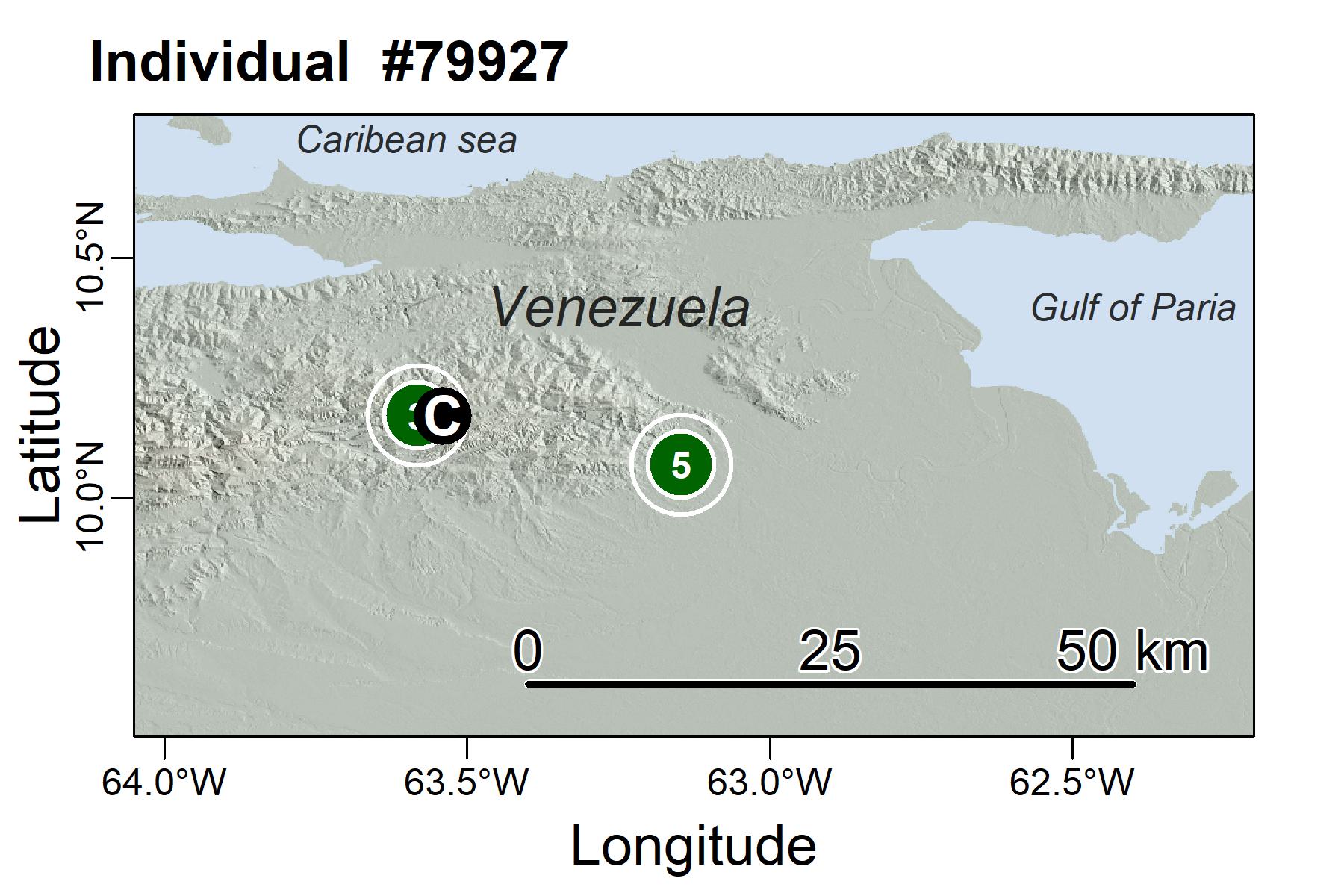


**Figure S14.** Site fidelity single individual to multiple foraging areas during the sampling period (Oilbird #79927, 61 GPS locations over 9 weeks), the numbers in the green circles are nights (revisits) at each site. C in a black circle represents the tagging site, Cueva del guacharo.


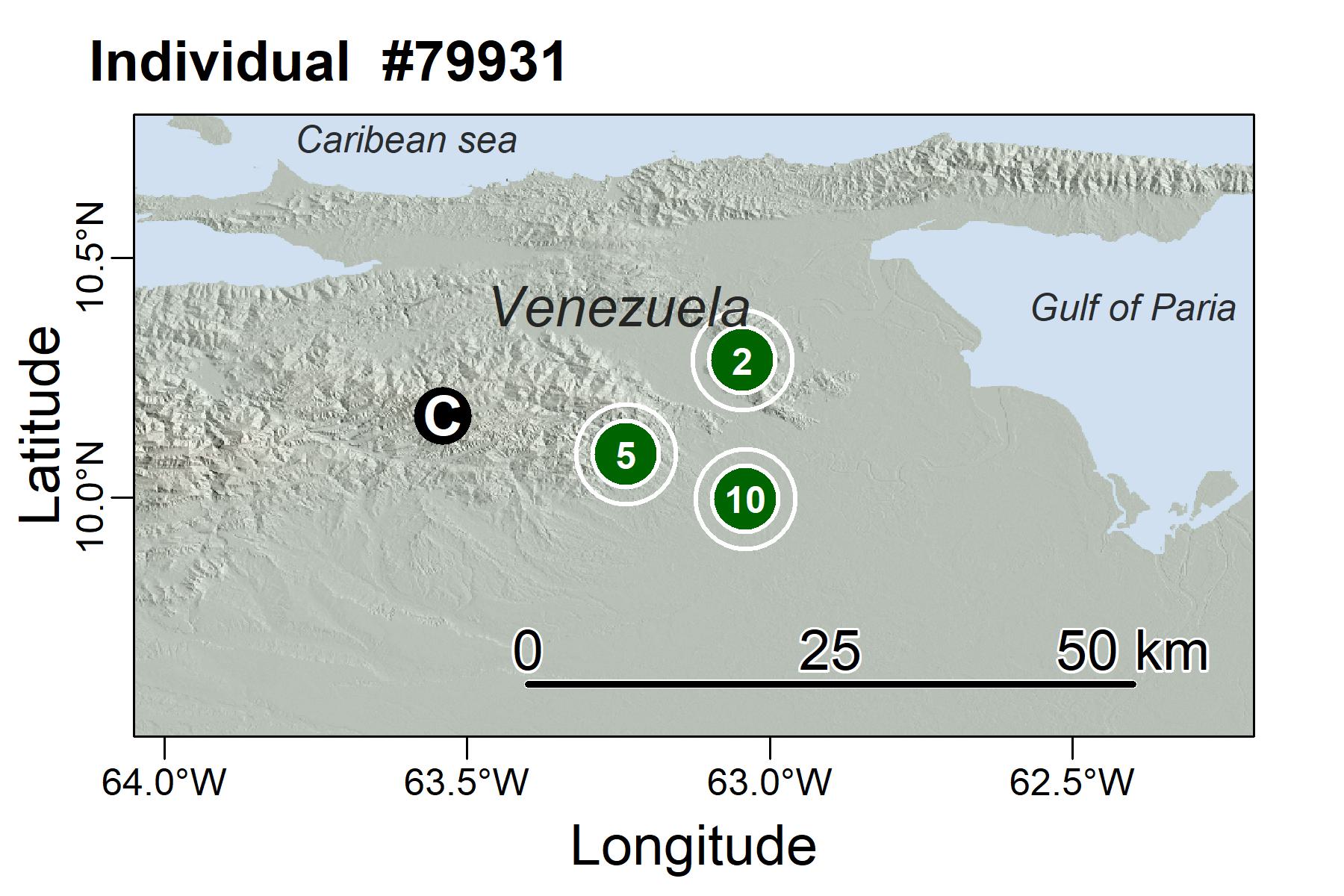


**Figure S15.** Site fidelity single individual to multiple foraging areas during the sampling period (Oilbird #79931, 127 GPS locations over 15 weeks), the numbers in the green circles are nights (revisits) at each site. C in a black circle represents the tagging site, Cueva del guacharo.


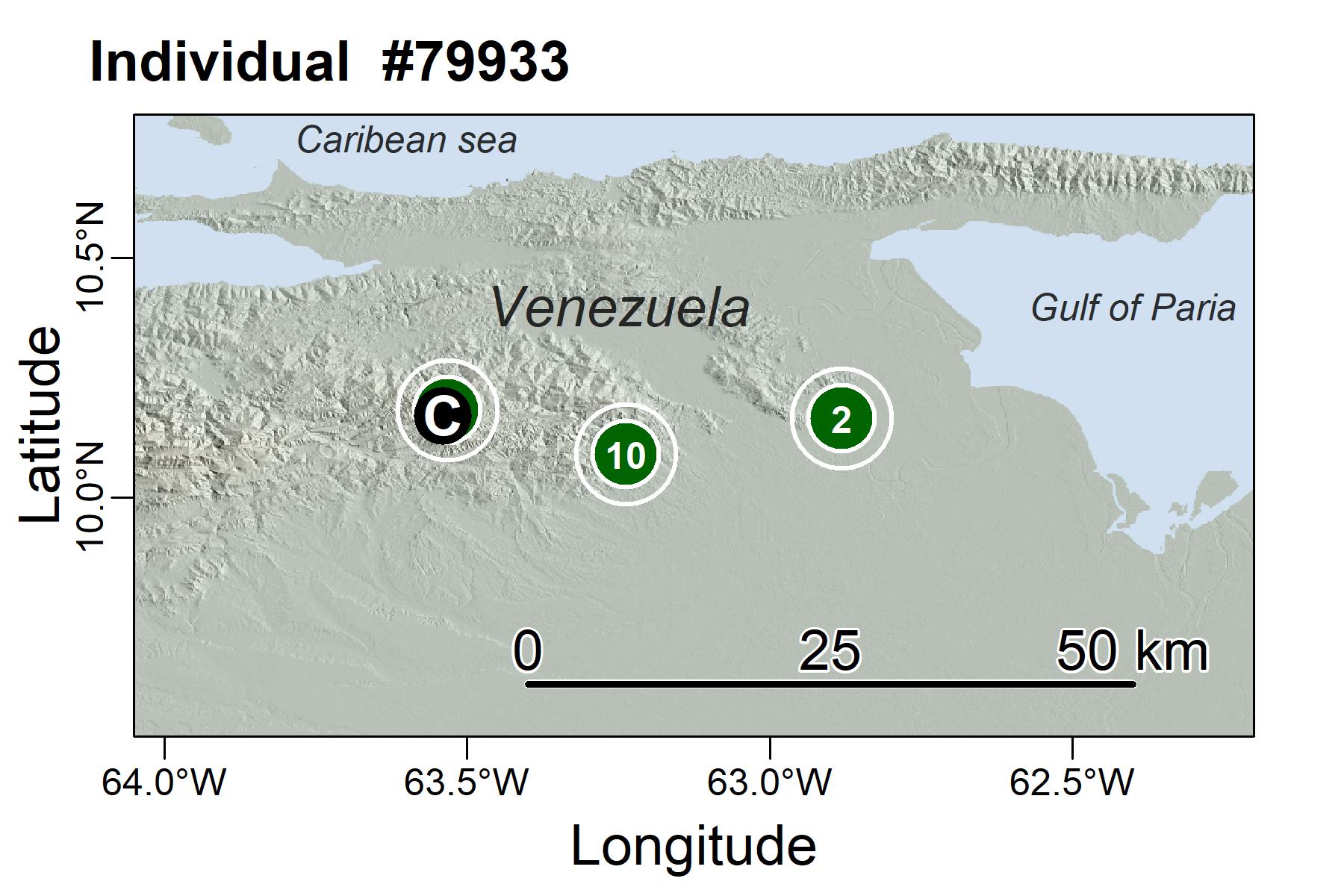


**Figure S16.** Site fidelity single individual to multiple foraging areas during the sampling period (Oilbird #79933, 133 GPS locations over 14 weeks), the numbers in the green circles are nights (revisits) at each site. C in a black circle represents the tagging site, Cueva del guacharo.


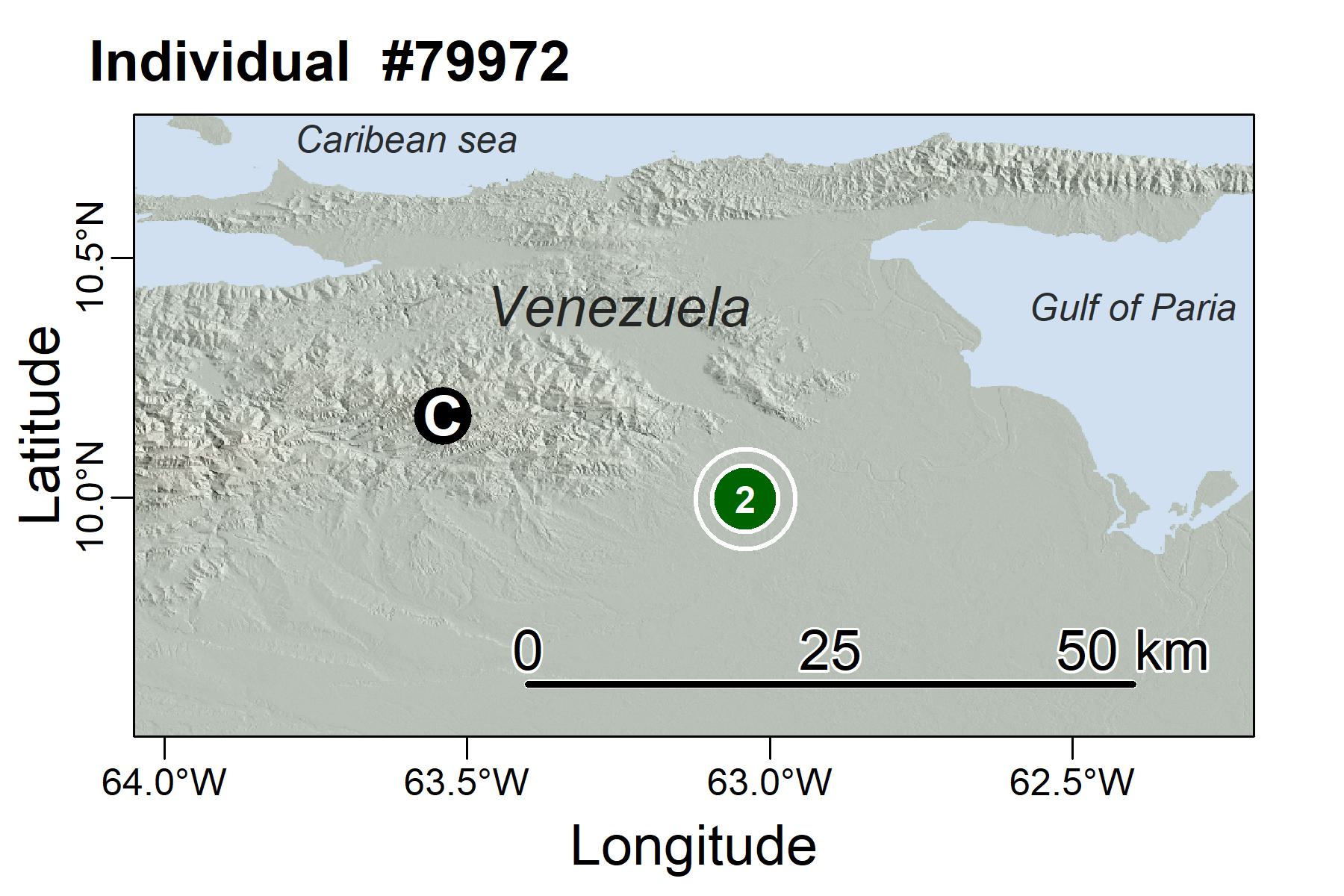


**Figure S17.** Site fidelity single individual to multiple foraging areas during the sampling period (Oilbird #79972, 57 GPS locations over 6 weeks), the numbers in the green circles are nights (revisits) at each site. Some areas are outside of the shown areas. C in a black circle represents the tagging site, Cueva del guacharo.


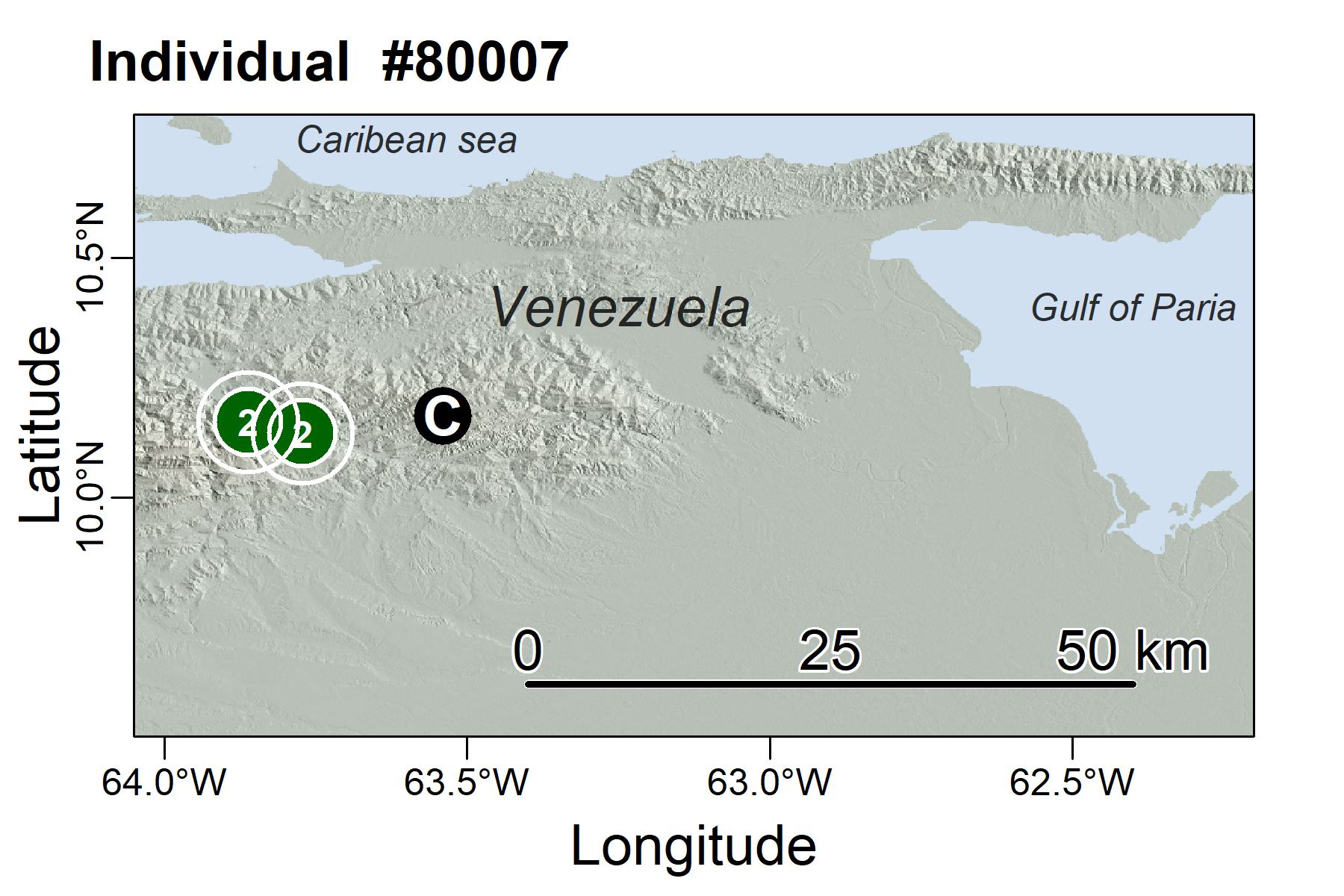


**Figure S18.** Site fidelity single individual to multiple foraging areas during the sampling period (Oilbird #80007, 33 GPS locations over 3 weeks), the numbers in the green circles are nights (revisits) at each site. C in a black circle represents the tagging site, Cueva del guacharo.


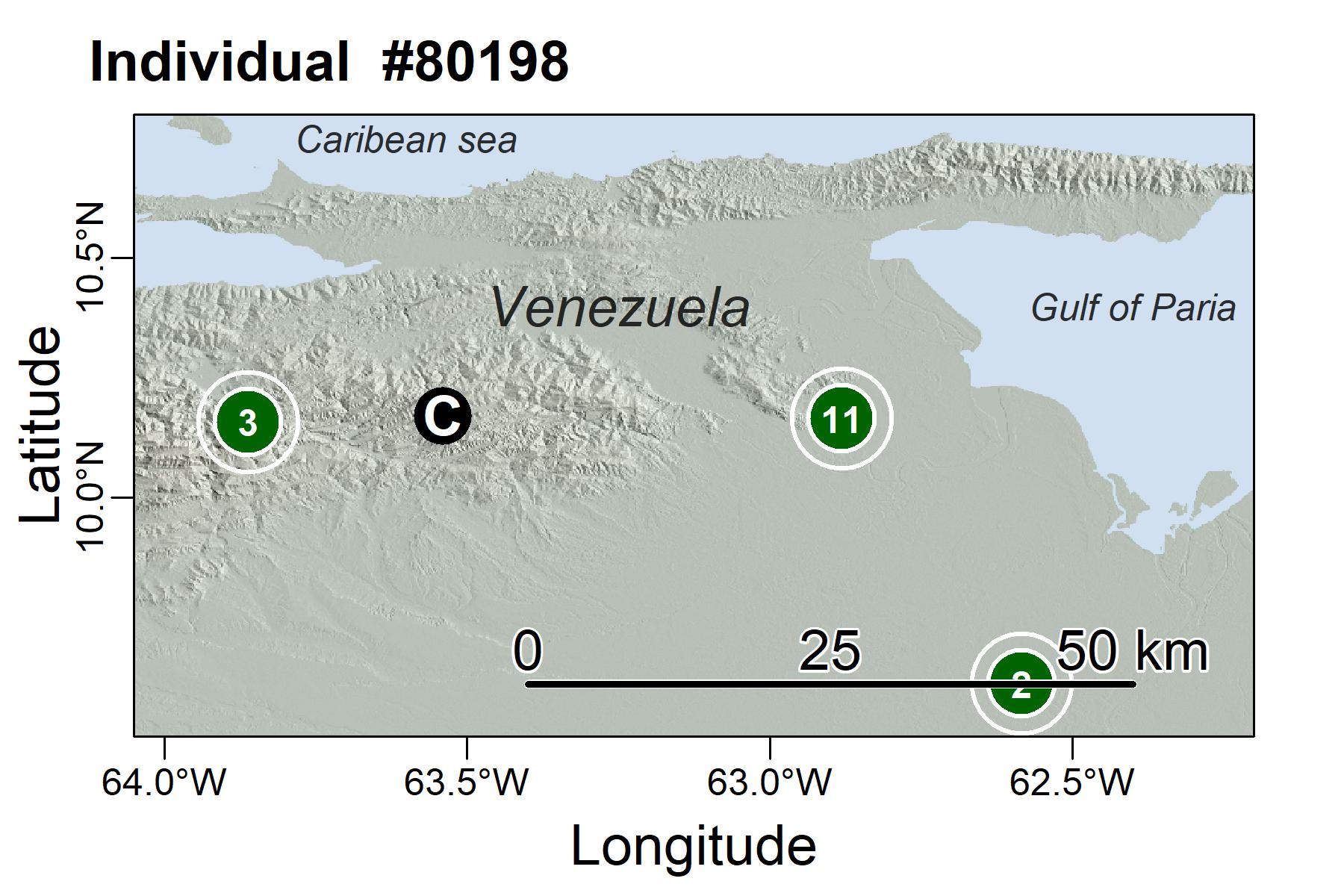


**Figure S19.** Site fidelity single individual to multiple foraging areas during the sampling period (Oilbird #80198, 169 GPS locations over 18 weeks), the numbers in the green circles are nights (revisits) at each site. C in a black circle represents the tagging site, Cueva del guacharo.


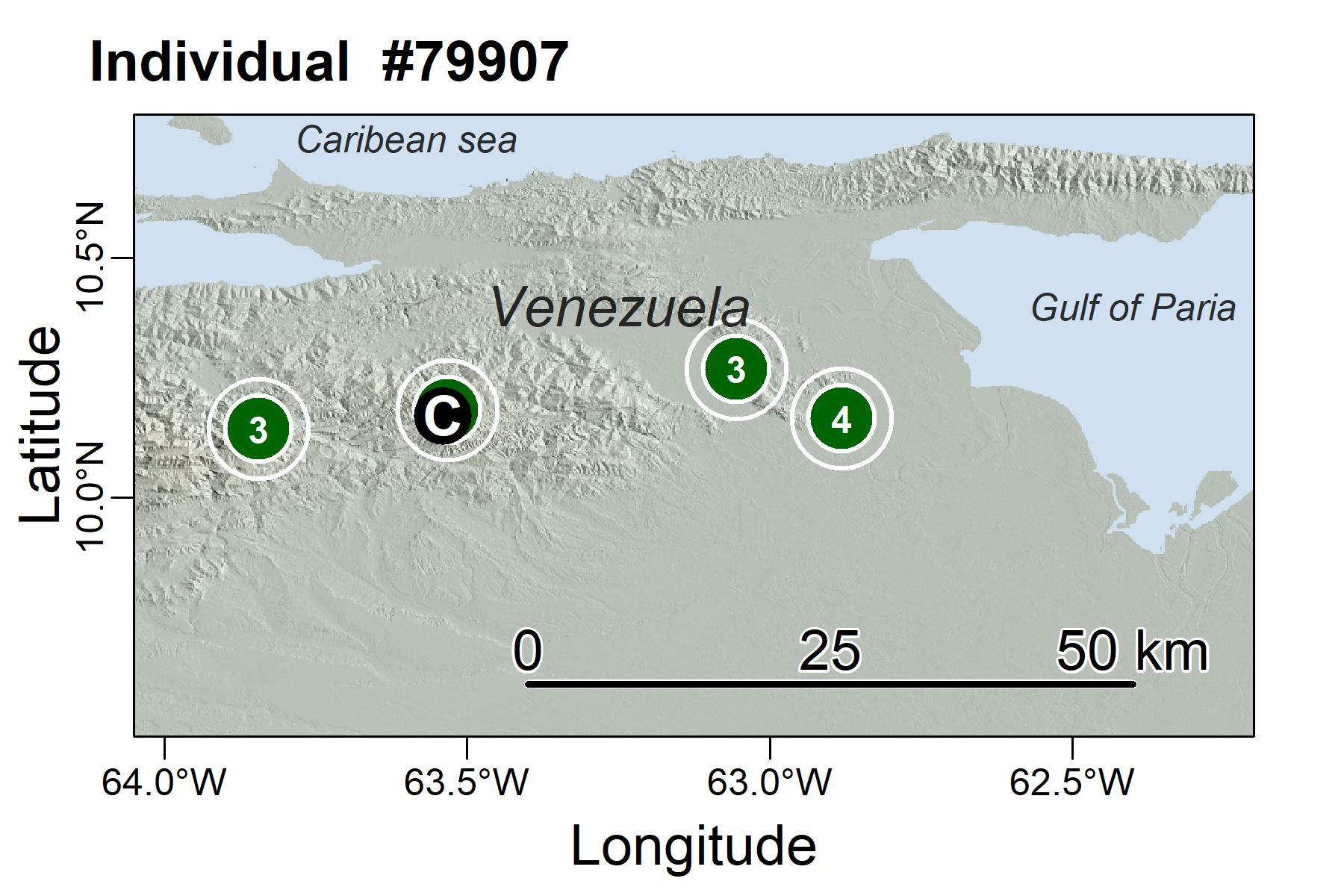


**Figure S20.** Site fidelity single individual to multiple foraging areas during the sampling period (Oilbird #79907, 108 GPS locations over 12 weeks), the numbers in the green circles are nights (revisits) at each site. C in a black circle represents the tagging site, Cueva del guacharo.


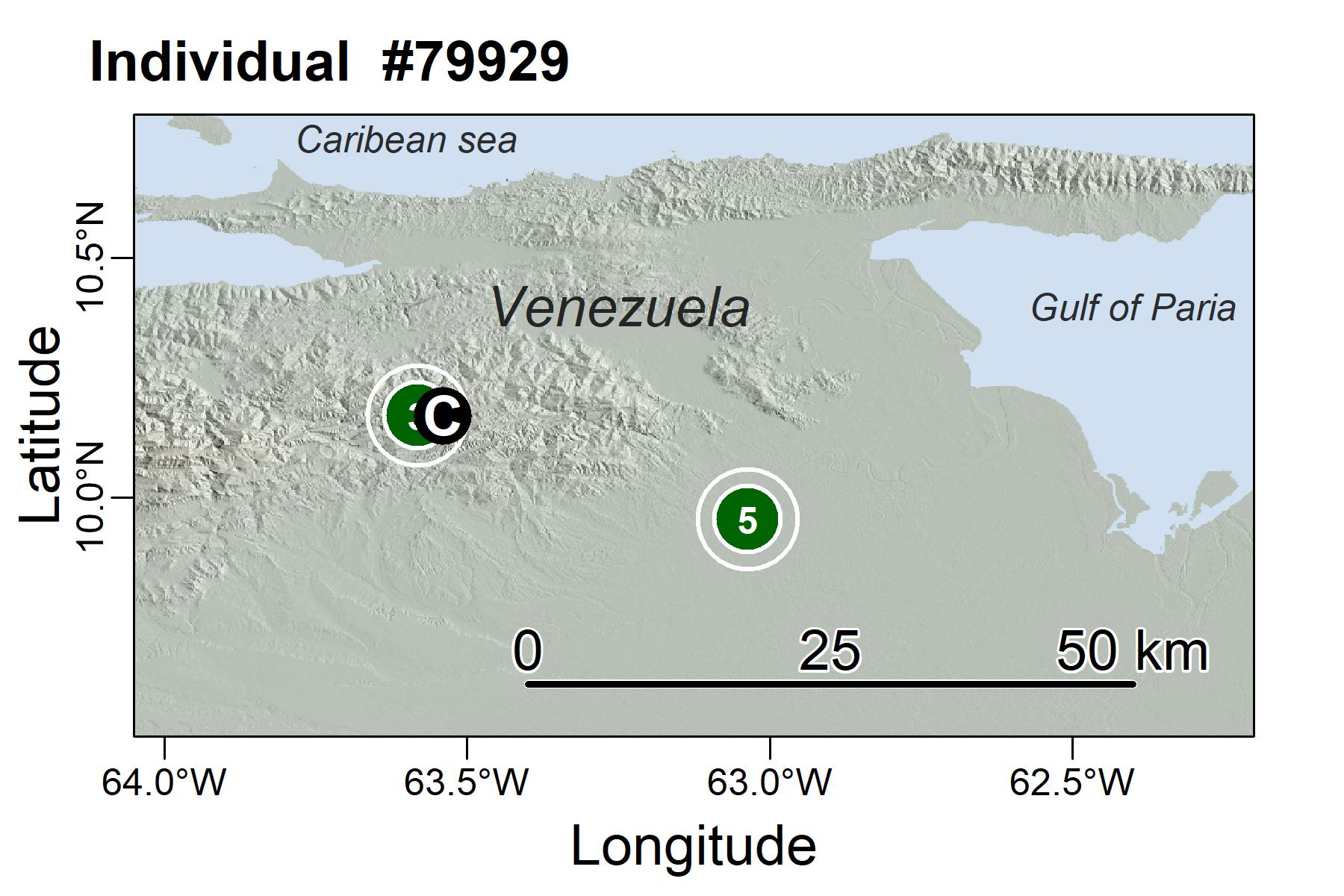


**Figure S21.** Site fidelity single individual to multiple foraging areas during the sampling period (Oilbird #79929, 126 GPS locations over 17 weeks), the numbers in the green circles are nights (revisits) at each site. C in a black circle represents the tagging site, Cueva del guacharo.


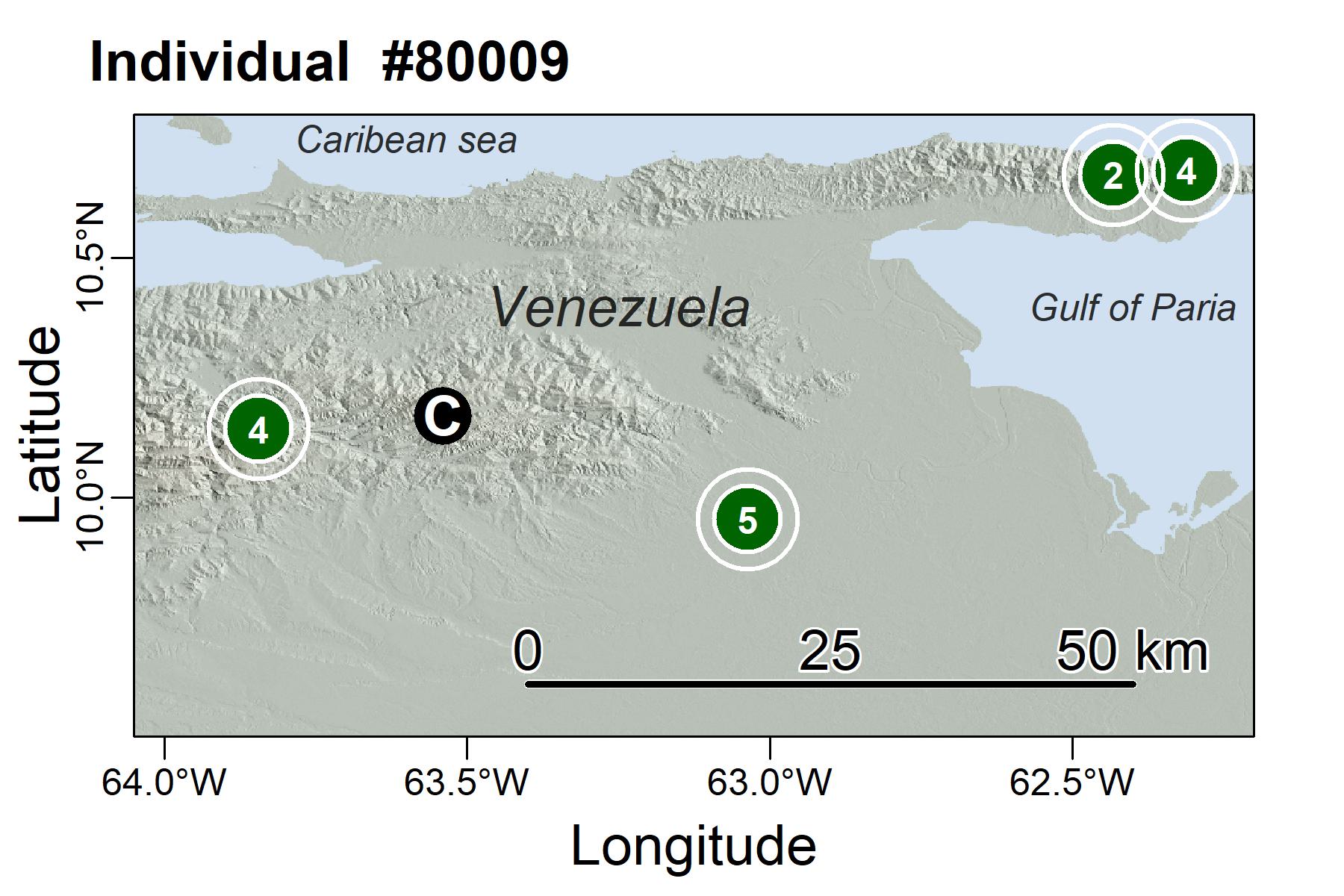


**Figure S22.** Site fidelity single individual to multiple foraging areas during the sampling period (Oilbird #80009, 133 GPS locations over 14 weeks), the numbers in the green circles are nights (revisits) at each site. C in a black circle represents the tagging site, Cueva del guacharo.


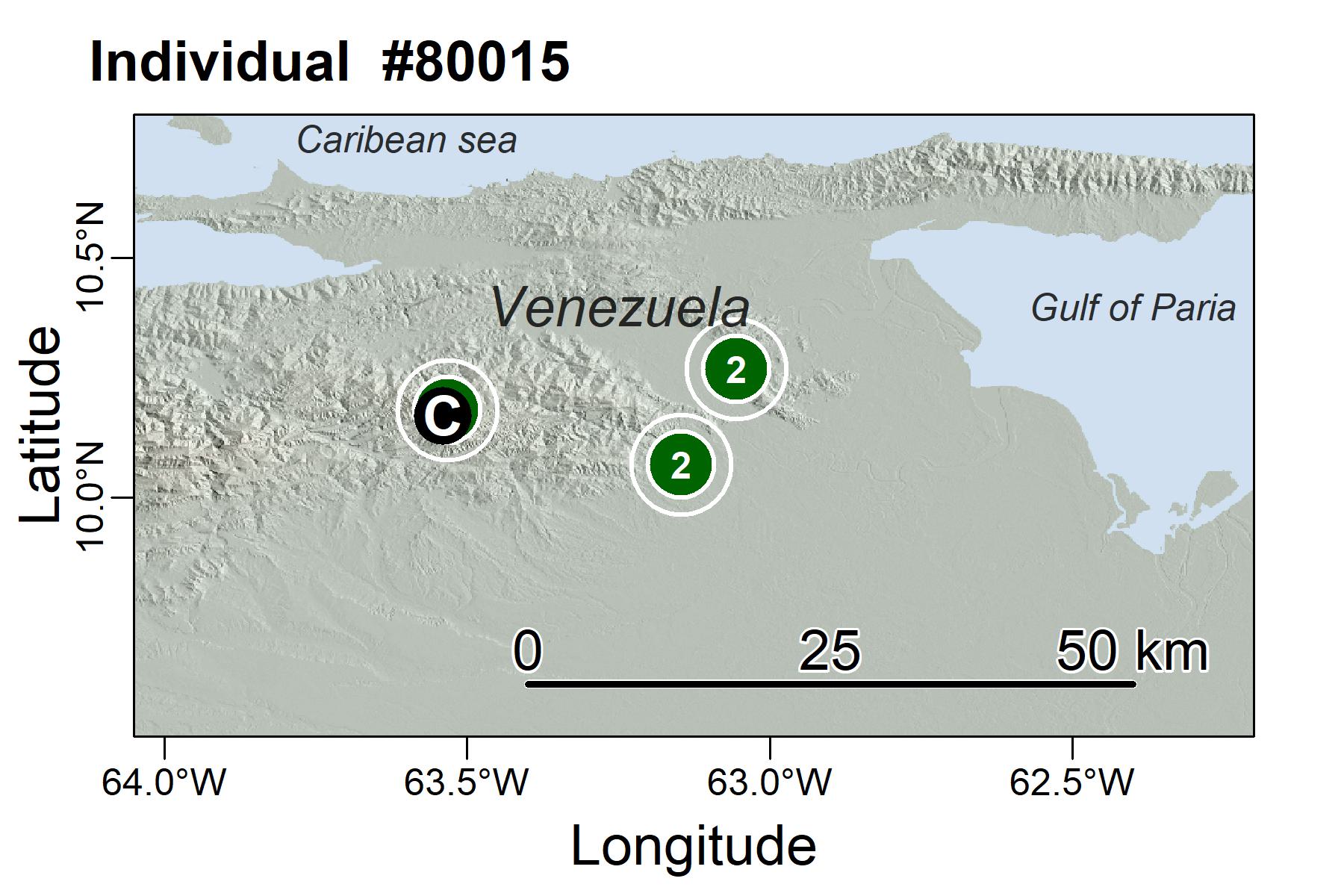


**Figure S23.** Site fidelity single individual to multiple foraging areas during the sampling period (Oilbird #80015, 61 GPS locations over 6 weeks), the numbers in the green circles are nights (revisits) at each site. C in a black circle represents the tagging site, Cueva del guacharo.


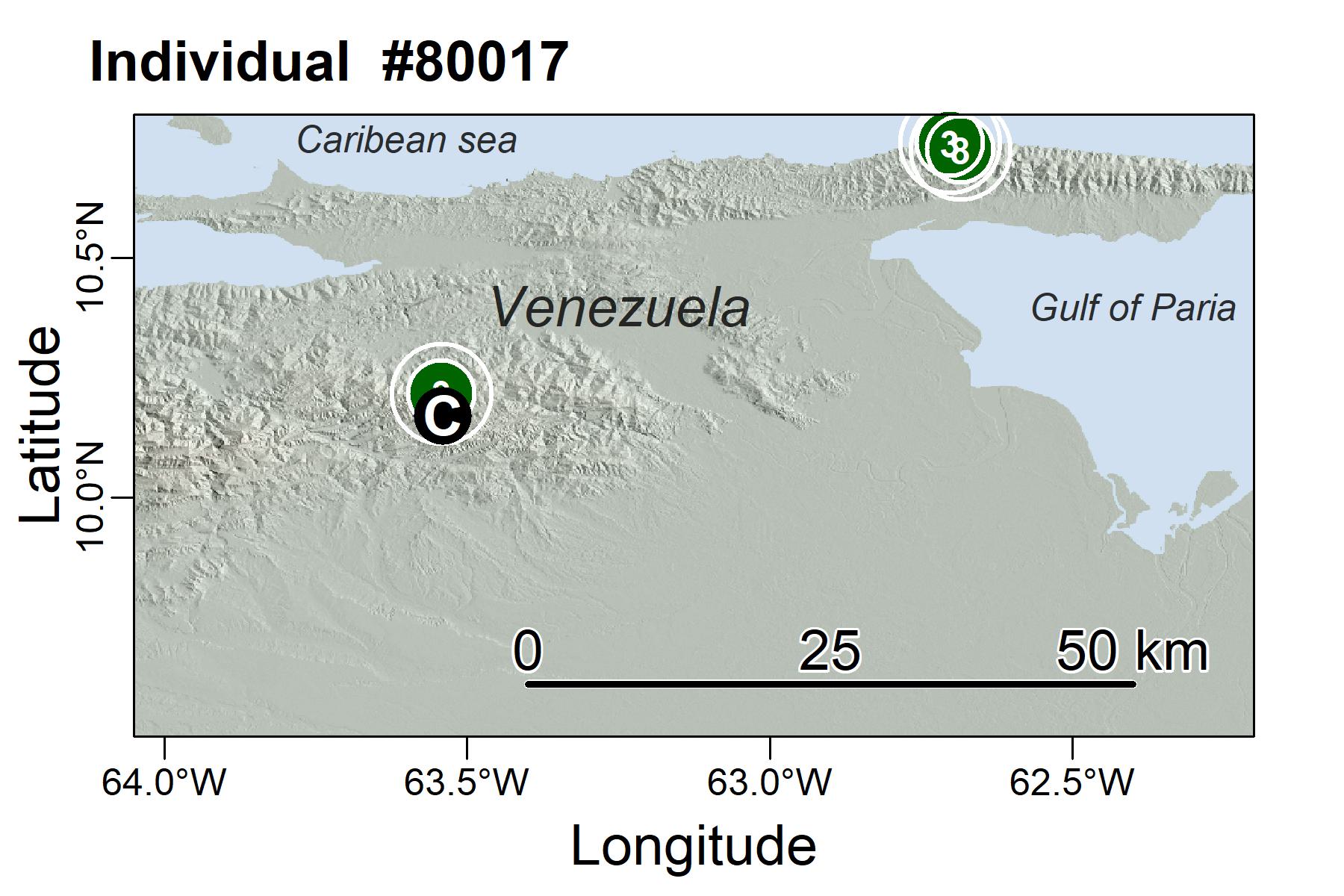


**Figure S24.** Site fidelity single individual to multiple foraging areas during the sampling period (Oilbird #80017, 108 GPS locations over 18 weeks), the numbers in the green circles are nights (revisits) at each site. C in a black circle represents the tagging site, Cueva del guacharo.
